# Epstein–Barr Virus, But Not Human Papillomavirus, Is Associated With Preinvasive and Invasive Ocular Surface Squamous Neoplasias in Zambian Patients

**DOI:** 10.3389/fonc.2022.864066

**Published:** 2022-04-14

**Authors:** Peter Julius, Stepfanie N. Siyumbwa, Phyllis Moonga, Fred Maate, Trevor Kaile, Gleb Haynatski, Veenu Minhas, Jazmine Snow, Kerstin Peterson, Patience Gihozo, Sam Streeter, Salan Kaur, Annika Evans, Daniela Gonzalez, Kandali Samwel, Guobin Kang, John T. West, Charles Wood, Peter C. Angeletti

**Affiliations:** ^1^ Department of Pathology and Microbiology, School of Medicine, Lusaka, Zambia; ^2^ University Teaching Hospital, Eye Hospital, Lusaka, Zambia; ^3^ Department of Biostatistics, University of Nebraska Medical Center, Omaha, NE, United States; ^4^ Nebraska Center for Virology and the School of Biological Sciences, University of Nebraska-Lincoln, Lincoln, NE, United States; ^5^ Ocean Road Cancer Institute, Dar es Salaam, Tanzania; ^6^ Department of Interdisciplinary Oncology, Louisiana State University Health Science Center, New Orleans, LA, United States

**Keywords:** ocular surface squamous neoplasia, human papillomavirus, Epstein–Barr virus, human immunodeficiency virus, Zambia

## Abstract

**Background:**

The etiopathogenesis of ocular surface squamous neoplasia (OSSN) is not fully understood. We assessed the frequency of oncogenic viruses in OSSN by immunohistochemistry (IHC) and polymerase chain reaction (PCR) for human papillomavirus (HPV), Epstein–Barr virus (EBV), Merkel cell polyomavirus (MCPyV), Kaposi sarcoma virus, and adenovirus. Cases from Zambia were prospectively enrolled using a cross-sectional study design between November 2017 and March 2020.

**Methods:**

Demographic and clinical data [age, sex, HIV status, antiretroviral therapy (ART) history, CD4 count, plasma viral load] and tumor biopsies were collected from 243 consenting patients. Tumor samples were bisected, and half was used for DNA isolation, while the other half was formalin fixed and paraffin embedded (FFPE) for histopathology analysis. The expressions of latent EBV nuclear antigen 1 (EBNA1), CDKN2A/p16INK4A (p16), and MCPyV large T-antigen (LT) were tested by IHC. Multiplex PCR was used to detect 16 HPV genotypes and four other DNA tumor viruses [Kaposi’s sarcoma-associated herpesvirus (KSHV), EBV, MCPyV, and adenovirus]. Relationships between HIV status, viral DNA and protein expression, and tumor grades were determined by statistical analysis.

**Results:**

OSSN tumors from patients were 29.6% preinvasive and 70.4% invasive. Patients presented with unilateral tumors that were 70.4% late stage (T3/T4). OSSN patients were HIV positive (72.8%). IHC on 243 FFPE biopsies resulted in the detection of EBNA1 (EBV), p16 high-risk HPV (HR-HPV), and MCPyV LT expression in 89.0%, 4.9%, and 0.0%, respectively. EBNA1 was expressed in all grades of preinvasive [cornea–conjunctiva intraepithelial neoplasia (CIN)1, 100%; CIN2, 85.7%; CIN3, 95.8%; and carcinoma *in situ* (CIS), 83.8%] and in invasive (89.2%) OSSN. PCR on 178 samples detected EBV, HR-HPV, and MCPyV in 80.3%, 9.0%, and 13.5% of tumors, respectively. EBV was detected in all grades of preinvasive and invasive OSSN. EBV detection was associated with high HIV viral loads (p = 0.022). HR-HPV was detected in 0.0% CIN1, 0.0% CIN2, 5.6% CIN3, 13.0% CIS, and 7.0% invasive OSSN.

**Conclusions:**

Our findings of EBV DNA and EBNA1 protein in all the grades of preinvasive and especially invasive OSSN are consistent with a potential causal role for EBV in OSSN. A role of HPV in OSSN was not clearly established in this study.

## Introduction

Ocular surface squamous neoplasia (OSSN) is the most common malignancy of the ocular surface (cornea and conjunctiva) (1). The reported increase in OSSN incidence is mainly due to the surge in the number of cases reported from sub-Saharan Africa, which are associated with HIV infection (2) and compounded by limited access to healthcare, infectious diseases, and poverty (3).

The etiology of OSSN remains unclear and may be multifactorial (4). UV-B radiation has been shown to induce OSSN (1, 5–10). Immunosuppression and chronic inflammation resulting from HIV infection may promote OSSN development (1, 11). HIV-infected persons are more likely to be infected by other infectious agents, including oncogenic viruses, than the general population (12–14) and have a 12-fold increased risk for OSSN (2). Oncogenic viruses linked to HIV-associated malignancy include Epstein–Barr virus (EBV), associated with nasopharyngeal carcinoma, Hodgkin’s and non-Hodgkin’s lymphoma and human papillomavirus (HPV), associated with carcinomas of the cervix, anus, penis, oropharynx, vulva, and vagina, Kaposi sarcoma virus, Kaposi’s sarcoma-associated herpesvirus (KSHV), pleural effusion lymphoma, multicentric Castleman disease, Merkel cell polyomavirus (MCPyV) associated with Merkel cell carcinoma, and hepatitis B and C viruses (HBV/HCV) (liver cancer) (15–17).

Immunosuppression reduces surveillance for infections and tumor cells, resulting in reactivation or persistence of disease, and/or chronic inflammation, which increases the risk for malignant transformation (18). The observation that OSSN develops in younger patients with HIV/AIDS raises the question about whether commonly acquired viruses are associated with OSSN.

HPV is an epitheliotropic DNA virus that causes malignancy at multiple mucosal sites in the body (19). The IARC has estimated that HPV is associated with 8.6% and 0.8% of solid tumors in women and men, respectively (4, 19). Several studies have investigated the role of HPV in the etiology of preinvasive (cornea–conjunctiva intraepithelial neoplasia) and invasive [cornea–conjunctiva squamous cell carcinoma (CSCC)] OSSN as well as uveal cancers (20). They have reported variation in prevalence rates ranging from 0% to 100% (20). Recently, in a systematic review of 39 studies, Ramberg et al. (20) found a pooled HPV prevalence of 26% in OSSN, with the lowest frequencies reported in African countries (21–25).

The low frequency and variation in HPV prevalence may be due to the small sample size used in most studies (20), use of archived formalin-fixed and paraffin-embedded (FFPE) blocks with suboptimal tissue preservation for molecular studies, clinical heterogeneity of tumors, and/or inadequate sampling of tumors for molecular testing. The World Health Organization’s (WHO) International Agency for Research on Cancer (IARC) has concluded that the evidence that HPV, as an etiologic agent for OSSN, is weak (4).

Using optimal and consistently preserved FFPE tissue, and fresh frozen tissue samples, the present study aimed to investigate the prevalence of different oncogenic viruses, EBV, HPV, MCPyV, KSHV, and adenoviruses in preinvasive and invasive OSSN. Also, we wanted to determine the relationship between the patient clinicopathologic features and the oncogenic virus status of the tumor tissue.

## Methods

**Figure 1:**
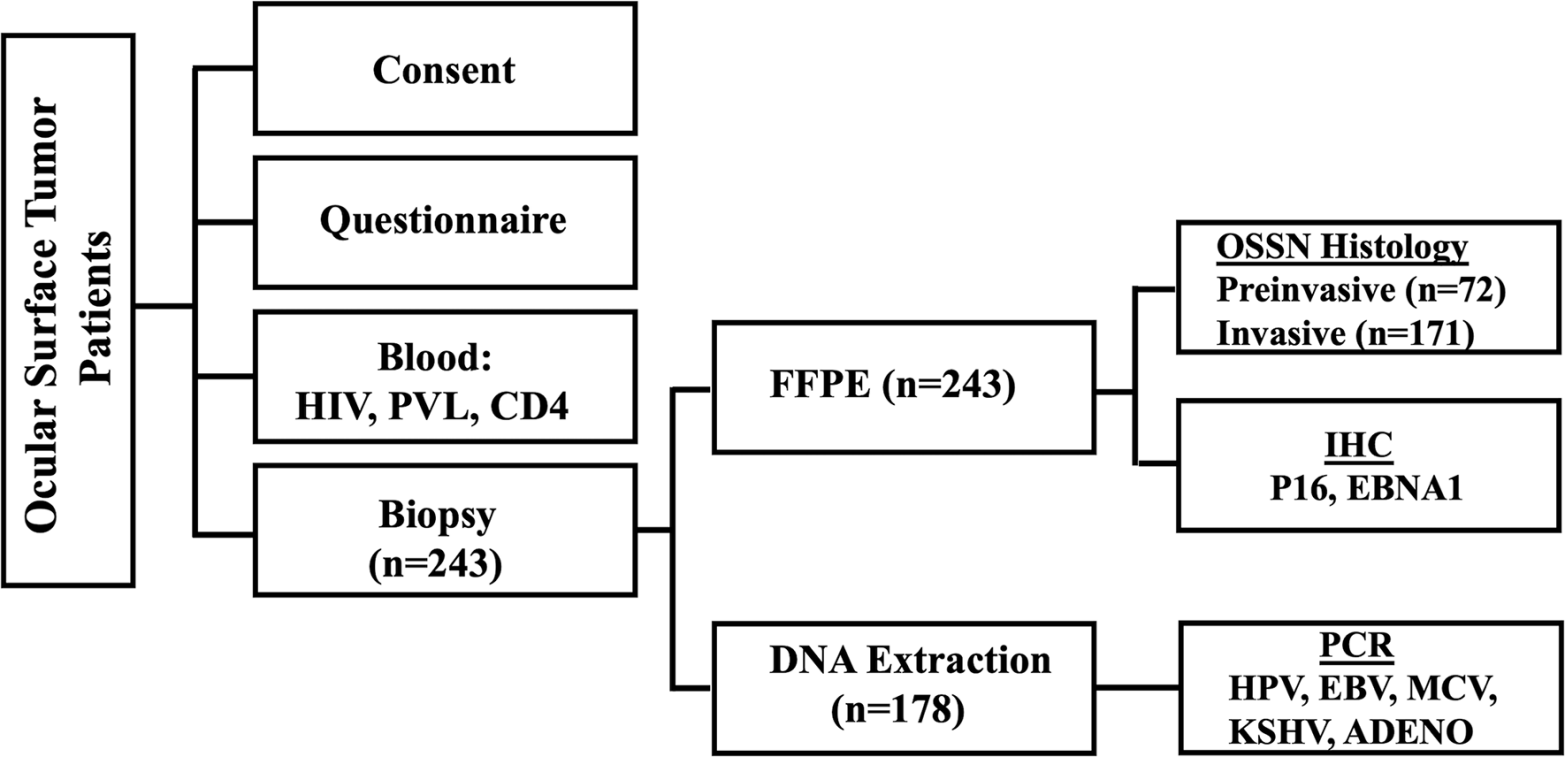
Experimental design.

The University of Zambia Biomedical Research Ethics Committee (UNZABREC IRB # 015-05-17), the Zambia National Health Research Authority, and the University of Nebraska-Lincoln’s Institutional Review Board (IRB # 20170817442FB) approved this study. Using a cross-sectional study design (**Figure 1**), we prospectively recruited patients 18 years and older who presented with an ocular surface tumor at the University Teaching Hospital in Lusaka, Zambia, between November 2017 and March 2020, as previously described (26). Consenting patients with a diagnosis of preinvasive and invasive OSSN and with adequate FFPE tissue for immunohistochemistry (IHC) were included in this analysis.

Data on age, sex, HIV status, antiretroviral therapy (ART) history, CD4 count, plasma viral load, diagnostic category, tumor subtype, grade, and stage were collected. All participants had primary disease with no previous treatments received. Clinical staging of the tumors followed the eighth edition of the American Joint Committee on Cancer Staging manual (2018) (27). Following clinical assessment and histologic confirmation of OSSN diagnosis, tumor samples were collected and processed as previously described (26). Two pathologists reviewed and examined hematoxylin and eosin (H&E)- and mucicarmine-stained slides. The final diagnosis was arrived at independently and, when they differed, by consensus. We classified tumors into preinvasive [cornea–conjunctiva intraepithelial neoplasia (CIN)] and invasive (CSCC) OSSN according to the fourth edition of the WHO classification of tumors of the eye (28). The preinvasive tumors were subtyped as CIN1, CIN2, and CIN3 and carcinoma *in situ* (CIS). Invasive OSSNs were subtyped and graded according to the WHO Classification of Tumors of the Eye, fourth edition (28). Vascular and perineural invasion was assessed for each invasive tumor.

A total of 243 patient FFPE tissue blocks with adequate tumor tissue were included in the analysis. One hundred seventy-eight patients had accompanying tissue for nucleic acid extraction, fixed in RNAlater solution for 24 h, subsequently kept at -80°C for long-term storage and subsequent analyses. The FFPE and frozen tissue samples were shipped to the University of Nebraska for IHC, DNA extraction, and PCR amplification.

### Immunohistochemistry

IHC was performed on FFPE tissue sections to analyze the expression of latent Epstein–Barr virus nuclear antigen 1 (EBNA1), CDKN2A/p16INK4A (p16), and MCPyV LT to facilitate the detection of EBV, HR-HPV, and MCPyV tumor viruses, respectively. The antibodies and dilutions used for the IHC analysis were as follows: anti-EBV nuclear antigen/EBNA1 mouse monoclonal antibody [E1-2.5] (ab8329, Abcam, USA) was used at a 1/50 dilution, anti-CDKN2A/p16INK4A mouse monoclonal antibody [JC2] (ab267833, Abcam USA) was used at a 1/1,000 dilution, and anti-MCPyV LT mouse monoclonal antibody [2t2] (MABF2316, EMD Millipore, USA) was used at a 1/500 dilution.

Briefly, 6-µm-thick tissue sections were cut from the tissue blocks and mounted on charged slides. The slides were incubated in a 60°C oven overnight. The tissue sections were deparaffinized in two subsequent washes (5 min each) using xylene and rehydrated by 5-min subsequent washes in 100%, 100%, 85%, and 70% ethanol. Endogenous peroxidase activity was blocked using 3% hydrogen peroxide (H_2_O_2_) methanol solution for 30 min at room temperature. Slides were rinsed in distilled water (three changes at 3 min each). Antigen retrieval was achieved by boiling in 10 mM citrate buffer, pH 6, for 15 min using a steamer. Slides were allowed to cool in the buffer for 20 min. The tissue was rinsed using a 1× phosphate-buffered saline (PBS) buffer. Normal goat serum (10%) was used as a blocking solution for 30 min at room temperature in a humidity chamber. Slides with primary antibodies were incubated overnight at 4°C in a humidity chamber. Following overnight incubation, slides were allowed to warm up to room temperature for 1 h and rinsed in 1× PBS (three changes at 3 min each). Tissue slides were then incubated with the Anti-mouse horse radish peroxidase (HRP)-labeled secondary antibody (K4001, Dako, USA) for 30 min at room temperature. Slides were rinsed in 1× PBS (three changes at 3 min each). The signal was developed using the diaminobenzidine (DAB) substrate-Chromogen System (K3468, Dako, USA). Harris hematoxylin was used as a counterstain.

The stained slides were digitalized using a slide scanner (MoticEasyScan Pro 6, Motic, USA) and examined using the Motic digital slide assistant software (Motic DSAssistant VM 3.0). A strong and diffuse nuclear and cytoplasmic staining by p16, a surrogate marker for high-risk HPV (HR-HPV) infection, in more than 75% of the tumor cells was considered positive for HR-HPV infection. Intense punctate or diffuse staining within the nucleus by anti-EBV nuclear antigen/EBNA1 antibody IHC was deemed positive for EBV infection. Perinuclear dot staining by anti-MCPyV LT antibody was considered positive for MCPyV infection.

### DNA Extraction From Ocular Surface Squamous Neoplasia Samples

According to the manufacturer’s instructions, DNA extraction from OSSN samples was carried out. DNA was extracted from the frozen tumor tissue using the Qiagen DNeasy Blood and tissue kit (Qiagen Inc., Valencia, CA, USA; catalog number 69506). The concentrations of the extracted genomic DNA were determined using a nanodrop spectrophotometer. DNA samples were stored at -20°C until PCR analysis. PCR amplification of the beta-globin gene was used to assess the quality of the extracted DNA. The beta-globin primers used are included in **Supplementary Table S5**.

### HPV Multiplex PCR Assay

PCRs for 16 HPV genotypes (6, 11, 16, 18, 30, 31, 33, 35, 39, 45, 51, 52, 56, 58, 59, and 66) were performed using a multiplex PCR kit. The PCR was performed in a single reaction tube using a type-specific Multiplex PCR kit (Qiagen Inc., Redwood City, CA, USA), following the manufacturer’s instructions and as described previously (29, 30). One microliter of DNA sample solution containing at least 50 ng of DNA (clinical sample or HPV plasmid DNA as a positive control) was used as the template for PCR amplification. The thermocycler conditions included incubation at 95°C for 15 min, 40 cycles of denaturation at 94°C for 30 s each, annealing at 70°C for 90 s, extension at 72°C for 60 s, and final extension at 72°C for 60 s. The PCR products were analyzed on a 5% polyacrylamide gel electrophoresis in 1× tris-borate ethylenediamine tetraacetic acid (TBE) and stained with ethidium bromide. The gel images were captured by the ChemiDoc MP Imaging system (Bio-Rad, Hercules, CA, USA). A sample was called positive for a specific HPV genotype when a clear band was visualized on the gel. A single unique molecular weight band detected for each HPV genotype, except for HPV16, which was detected by visualization of two separate bands, as a means of confirmation. The detection limit was between 1 and 10 copies per reaction (29, 30).

### DNA Tumor Virus Multiplex and Epstein–Barr Virus Confirmation PCR Assays

PCRs for four DNA tumor viruses (KSHV, EBV, MCPyV, and adenovirus) were performed using a multiplex PCR kit using the primers indicated in **Supplementary Table S5** in a single reaction. Primers for adenovirus were developed and evaluated by Ko et al. (31) and Xu et al. (32), while those for and KSHV by Mortazavi et al. (33). The primers for EBV and MCPyV for the multiplex PCR were designed and developed by this study.

DNA tumor virus (DNATV) PCR was performed in a single reaction tube using a Multiplex PCR kit (Qiagen Inc., Redwood City, CA, USA) following the manufacturer’s instructions. One microliter of DNA sample solution containing at least 50 ng of DNA of the clinical sample (or control DNA) was used as the template for PCR amplification. The thermocycler conditions included pre-melt at 94°C for 15 min, 40 cycles of denaturation at 94°C for 30 s each, annealing at 70°C for 90 s, extension at 72°C for 60 s, and final extension at 72°C for 10 min. The PCR products were analyzed on a 6% polyacrylamide gel electrophoresis in 1× TBE and stained with ethidium bromide. The gel images were captured by the ChemiDoc MP Imaging system (Bio-Rad, Hercules, CA, USA). A sample was called positive for a specific DNA tumor virus when a clear band was visualized on the gel. A single band of unique molecular weight was detected for each analyzed DNA tumor virus.

Representative samples from the EBV-positive and all the EBV-negative cases as determined by multiplex PCR were confirmed using EBV primers described previously (34, 35). The EBV confirmation primers are indicated in **Supplementary Table S5**. Thermocycler conditions used included incubation at 95°C for 4 min, 40 cycles of denaturation at 95°C for 1 min each, annealing at 55°C for 1 min, extension at 72°C for 1 min, and final extension at 72°C for 3 min using 1 µl DNA sample containing at least 50 ng of clinical specimen or control EBV control DNA. The PCR product was analyzed on a 5% polyacrylamide gel electrophoresis in 1× TBE and stained with ethidium bromide. The gel images were captured by the ChemiDoc MP Imaging system (Bio-Rad, Hercules, CA, USA). A sample was called positive for EBNA1 when a single band was visualized on the gel at least the same intensity as the control. The detection limit of EBV in these assays was 10 copies per reaction (34).

### Data Analysis

Statistical analysis was performed using GraphPad prism 9, and IBM SPSS Statistics 26 was used for organization and cleaning data and obtaining the frequency counts for the categorical data. Individual viral PCRs (EBV, HPV, and MCPyV) and IHC (EBNA1 and p16 IHC) status data were analyzed as dependent variables and assessed for a relationship with independent variables such as age, sex, HIV status, ART uptake status, CD4 count, viral load, diagnostic category, tumor grade, subtype, and stage. The infectious agents in tissue samples were reported as frequency/total (percentage). Continuous variables with distributions that depart from normal (Shapiro–Wilk p < 0.05) were reported as medians with interquartile 25% and 75%. Those with normal distributions were reported as mean ± SD. The chi-square and Fisher’s exact probability tests were used to assess the association between IHC reactivity and clinicopathologic variables, all assumptions applied. A Student’s t-test was conducted on two normally distributed variables and reported a difference between means (95% confidence interval). A Mann–Whitney U test was conducted on variables with one or two non-normally distributed continuous variables. The p-value was recorded, and the Hodges–Lehmann estimate was used to get the confidence interval across the groups. During variable selection, it was determined that the study had an insufficient number of variables suitable for logistic regression (variables with a frequency of cases >10 and chi-square <0.20). A p-value <0.05 was considered statistically significant.

## Results

A total of 243 patients with primary OSSN were diagnosed by histopathology using H&E-stained slides (**Table 1**). Most (99.6%) of the patients had unilateral disease, with most tumors (54.5%) reported in the right eye. OSSN diagnoses comprised 72 (29.6%) preinvasive and 171 (70.4%) invasive tumors (**Figure 1**). Females predominated the study (61.3%) population. The frequency of preinvasive and invasive OSSN was similar in males and females (p = 0.091) (**Supplementary Table S4**). At diagnosis, the study population’s median age was 38.0 [interquartile range (IQR), 31.0–45.0) years overall (range: 18–76). At primary diagnosis, female patients presented at a significantly younger age (37.0 years; IQR, 29.0–43.0 years) than males (40.6 ± 9.9, p = 0.007). Preinvasive cancer patients were significantly younger (34.5 years; IQR, 28.5–43.0 years) than invasive (39.0 years; IQR, 34.0–46.0, years) OSSN patients (p = 0.003) (**Supplementary Table S4**).

**Table 1:** Clinicopathological characteristics of the participants and their tumors.

Variable	Frequency (%)
Age	38.0 (31.0 – 45.0) years
Sex	
Male	94/243 (38.7)
Female	149/243 (61.3)
HIV status	
Positive	177/243 (72.8)
Negative	66/243 (27.2)
Awareness HIV +	
Aware HIV+	163/177 (92.1)
Unaware HIV+	14/177 (7.9)
ART Uptake	
Yes	151/177 (85.3)
No	26/177 (14.7)
CD4 Count	234.0 (119.8 – 411.8) cells/microliter
CD4 Count category	
<200	70/160 (43.8)
>200	90/160 (56.2)
Plasma HIV Viral load	0.0 (0.0 – 181.0) copies/milliliter
Plasma HIV Viral load (c)	
<200	71/95 (74.7)
>200	24/95 (25.3)
Diagnosis	
Invasive OSSN	171 (70.4)
Pre-invasive OSSN	72 (29.6)
Preinvasive tumor grade	
CIN1	2 (2.8)
CIN2	8 (11.1)
CIN3	25 (34.7)
CIS	37 (51.4)
Grouped Preinvasive tumor grade	
CIN1 and 2	10 (13.9)
CIN3 and CIS	62 (86.1)
Invasive tumor subtype	
Keratinizing squamous cell carcinoma	161 (94.2)
Basaloid squamous cell carcinoma	6 (3.5)
Spindle squamous cell carcinoma	4 (2.3)
Invasive tumor grouped	
Keratinizing	161 (94.2)
Non-keratinizing	10 (5.8)
Grade	
Well, differentiated	21 (12.3)
Moderately differentiated	140 (81.9)
Poorly differentiated	10 (5.8)
AJCC Stage	
T1	38 (22.2)
T2	42 (24.6)
T3	88 (51.5)
T4	3 (1.8)

The majority (86.1%) of the preinvasive OSSN diagnoses were composed of CIS (51.4%) and CIN3 (34.7%). Invasive OSSN subtypes were composed of conventional (keratinizing) SCC (CSCC) (n = 161, 94.2%), basaloid SCC (n = 6, 3.5%), and spindle cell carcinoma (n = 4, 2.3%). Moderately differentiated (grade 2) tumors predominated (n = 140, 81.9%), while grade 1 and 3 tumors accounted for 12.3% and 5.8%, respectively. None of the patients had been previously treated with surgery, chemotherapy, immunotherapy, or radiotherapy. Clinical staging of the invasive tumors determined that 46.8% and 53.2% of the tumors were early (stage 1 and 2, T1/2)- and late (stage 3 and 4, T3/T4)-stage cancers, respectively. Patients with late-stage cancer at diagnosis were significantly older (42.0 vs. 37.0, p = 0.005) than those with early-stage disease (**Table 1**).

We collected blood samples from all the patients for HIV, CD4, and plasma HIV viral load (VL) testing at recruitment, described previously (26). Most patients tested HIV positive (n = 177, 72.8%) (**Table 1**). There was no difference in the frequency of HIV infection with respect to sex (p = 0.664), but there was a significant difference between HIV infection vs. age (p = 0.007) (**Supplementary Table S4**). The frequency of preinvasive and invasive OSSN diagnoses was similar in HIV-positive and -negative patients (p = 0.160). HIV infection was not associated with any histologic subtype of the invasive tumors (p = 0.730). However, HIV infection was significantly associated with late-stage disease at primary diagnosis (p = 0.024).

Of the 177 patients who tested HIV positive, 163 (92.1%) knew their HIV status (**Table 1**). Newly diagnosed patients accounted for 7.9% (n = 14) of the HIV-positive OSSN patients. Of the 163 patients living with HIV who knew their HIV status, 92.6% reported taking combined ART and were compliant. Only one (0.7%) patient reported defaulting on his or her treatment.

CD4 counts were determined in 90.4% (n = 160) of the HIV-positive patients. The median CD4 counts were 236.0 (IQR, 121.5–414.5) cells/μl overall. Patients diagnosed with invasive tumors had significantly lower CD4 counts than those diagnosed with preinvasive (203.5, IQR, 115.5–388.3 vs. 325.0, IQR, 136.0–524.0, respectively) (p = 0.025). CD4 counts less than 200 cells/μl and greater than 200 cells/μl accounted for 43.8% and 56.2% of the OSSN patients, respectively. The CD4 count category was not significantly associated with the diagnostic category (preinvasive vs. invasive) of the tumor (p = 0.071) or the tumor stage of the invasive disease (p = 0.413). Plasma VL was determined in 95 (53.7%) patients. Most (74.7%) patients were viral suppressed (VL ≤200 copies/ml). Patients with invasive OSSN had significantly lower viral loads than preinvasive cancer patients (p < 0.001). Plasma viral load was inversely proportional to the CD4 counts; however, this relationship was not significant (r^2^ = -0.172, p = 0.112).

### HPV Detection

**Figure 2:**
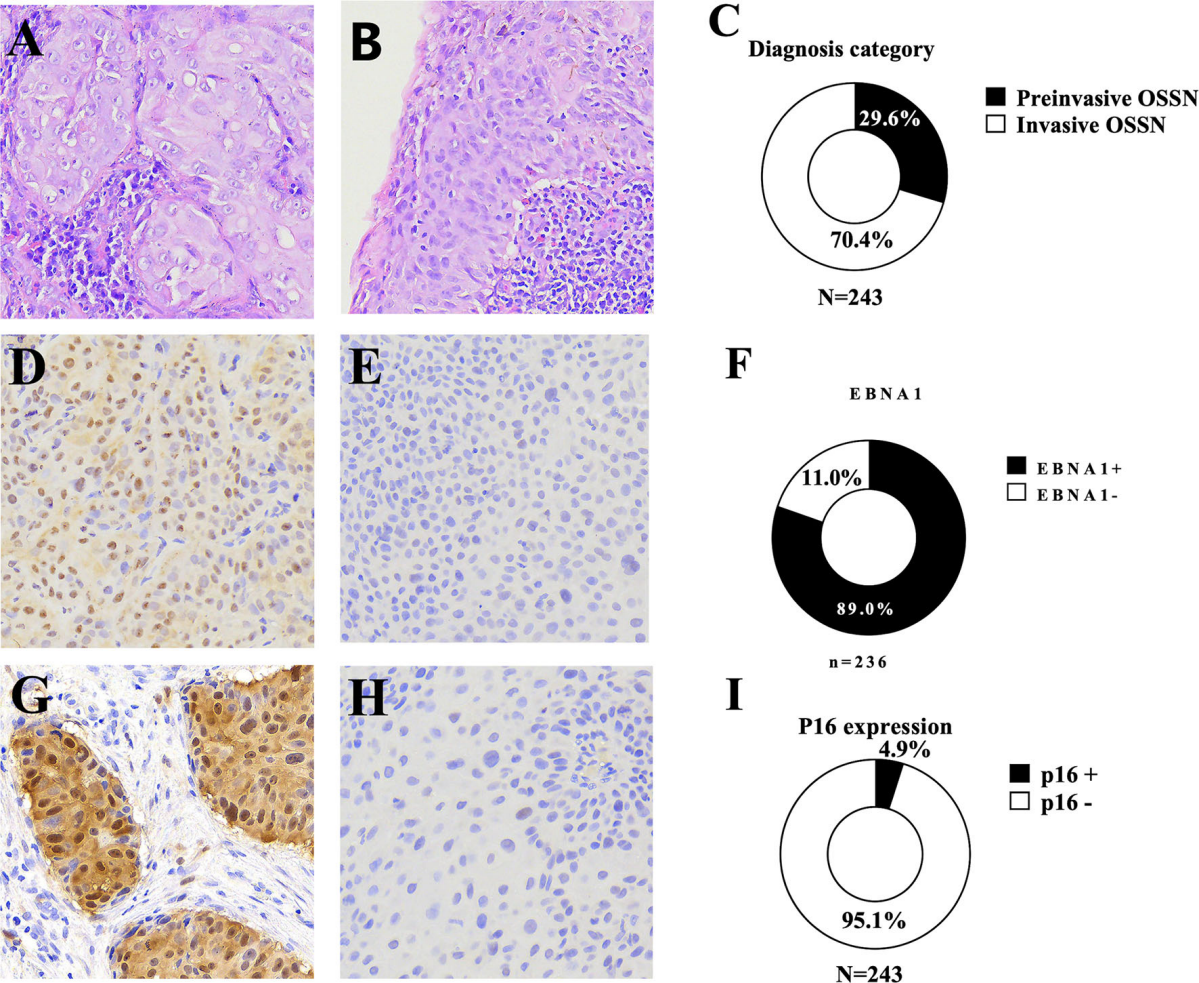
Representative microscopy recorded at ×20 magnification showing **(A)** non-keratinizing squamous cell carcinoma, **(B)** a preinvasive ocular surface squamous neoplasia [carcinoma *in situ* (CIS)] with inflammation in the underlying stroma and the overlying epithelium, **(C)** the frequency of preinvasive and invasive ocular surface squamous neoplasia, **(D)** an Epstein–Barr virus nuclear antigen 1 (EBNA1)-positive ocular surface squamous neoplasia [immunohistochemistry (IHC)], **(E)** EBNA1-negative ocular surface squamous neoplasia (IHC), **(F)** frequency of EBNA1 expression, **(G)** a p16-positive ocular surface squamous neoplasia, **(H)** a p16-negative ocular surface squamous neoplasia, **(I)** the frequency of P16 overexpression in ocular surface squamous neoplasia.

FFPE tissue samples from 243 patients were available for IHC testing. IHC staining for p16, a surrogate marker for HR-HPV infection (**Supplementary Table S1**), showed overexpression of p16 in 12 (4.9%) of the 243 patient samples tested. Partial expression and a complete lack of p16 were observed in 133 (54.7%) and 98 (40.3%) patient tissue samples, respectively, and these were considered negative for p16 overexpression. Of the 243 patients with FFPE tissue blocks, 178 (73.3%) had accompanying tissue collected and submerged in RNAlater solution for 24 h and stored at -80°C for subsequent DNA extraction and PCR amplification (**Figure 2**). DNA extracted from all the 178 tissue samples tested positive for cellular (beta-globin) marker and was thus suitable for virus PCR analyses. Using multiplex PCR, HPV was detected in 17 (9.6%) patient samples [16 HR-HPV, 9.0%, and one low-risk (LR)-HPV, 0.6%]. PCR analyses detected single and multiple HR-HPV genotypes in 10 (62.5%) and 6 (37.5%) cases, respectively. The most frequently detected HR-HPV genotypes were HPV33 (n = 6, 26.1%) followed by HPV 59 (n = 5, 21.7%) (**Table 2**, **Supplementary Figure S2**). We did not detect HPV16 in any of the samples analyzed. HPV18 was detected in three (13.0%) patient samples.

**Table 2:** Frequency of viral infection detection in tumor sample by PCR.

Virus tested for	Total Samples tested	PCR Result	
		Positive samples	Percentage
EBV	178	143	80.3
HR-HPV	178	16	9.0
MCPyV	178	24	13.5
KSHV	178	0	0
ADENOVIRUSES	178	0	0
CMV	178	0	0
Co-Infections			
EBV + HR-HPV	178	12	6.7
EBV + MCPyV	178	23	12.9
HR-HPV + MCPyV	178	7	3.9
EBV + MCPyV + HR-HPV	178	6	3.4

The frequency of HPV detection in preinvasive cancer was low (n = 4, 8.7%) (**Supplementary Table S2**). HPV was not detected in CIN1 and CIN2 lesions; however, it was found in one (5.6%) and three (13.0%) patients diagnosed with CIN3 and CIS, respectively. Similarly, p16 overexpression by IHC was not observed in any preinvasive tumors. However, p16 overexpression was present in 12 (7.0%) patient samples diagnosed with invasive OSSN (basaloid SCC, n = 2, 33.3% and CSCC, n = 10, 6.2%), while spindle cell carcinomas were negative. Of the 12 cases that showed p16 overexpression, three were positive for single HPV genotypes (HPV33, HPV58, and HPV59) and one was positive for multiple HPV genotypes (HPV45 and HPV51). One patient tumor diagnosed as CIN3 was positive for three HR-HPV genotypes (HPV31, HPV33, and HPV59); however, this case did not express p16 by IHC (**Table 2**). Six patient tumor samples with p16 overexpression had no detectable HPV. P16 overexpression was significantly associated with invasive (p = 0.020) OSSN category and grade 3 differentiation (poorly differentiated) in invasive tumors (p = 0.001) (**Supplementary Table S1**). A non-keratinizing invasive tumor was more likely to be positive for a HR-HPV infection (p = 0.036) (**Supplementary Table S1**) by PCR. We used p16 IHC and PCR to detect HPV DNA in OSSN, and both methods were consistent with a low frequency of HPV. We found that the test methods did not correlate well. IHC for p16 had a low (25%, CI: 0.102–0.475) sensitivity and a high (96.3%) specificity for predicting HR-HPV infection using multiplex HPV DNA PCR as a reference. The positive and negative predictive value of p16 for HR-HPV infection by PCR was 40.0% and 92.9%, respectively (**Supplementary Figure S1**). Also, the results showed that both molecular methods and IHC did not detect HR-HPV or p16 overexpression in early preinvasive OSSN lesions.

### MCPyV, CMV, KSHV, and Adenovirus Detection

Merkel cell virus (MCPyV) was detected in 24 (13.5%) patient samples by PCR (**Supplementary Table S3**). MCPyV was detected in 10.9% of preinvasive (n = 0, 0.0% CIN 1; n = 2, 66.7% CIN 3; n = 1, 5.6% CIN 3; and n = 1, 8.7% CIS) and in 14.4% of invasive (n = 17, 13.8% CSCC; n = 2, 33.3% basaloid SCC; and n = 0, 0.0% spindle cell carcinoma) OSSN. MCPyV positivity was not associated with any of the clinical and pathologic variables. None of the MCPyV patient samples that tested positive by PCR had detectable MCPyV LT by IHC on FFPE tissue blocks. The results show a low frequency of MCPyV DNA detected in preinvasive lesions and a lack of MCPyV viral protein expressed in OSSN lesions. PCR did not detect KSHV, CMV, or adenoviruses in patient tumor samples.

### EBV Detection

FFPE tissue samples from 236 patients were used for EBNA1 IHC analysis (**Table 3**). Two hundred ten (89.0%) primary tumor samples were positive for EBNA1 by IHC. EBNA1 protein was detected in 88.6% of preinvasive (n = 2, 100% CIN 1; n = 6, 85.7% CIN 2; n = 23, 95.8% CIN 3; and n = 31, 83.8% CIS) and in 89.2% of invasive (n = 142, 91.0% CSCC; n = 4, 66.7% basaloid SCC; and n = 2, 50% spindle cell carcinoma) OSSN. EBNA1 expression was significantly associated with a keratinizing SCC morphology (p = 0.002) and with moderate differentiation in invasive OSSN (p = 0.014). There was no significant association between EBNA1 positivity and level of immunity, invasiveness, dysplasia, and stage of invasive cancer (**Table 3**). We observed EBNA1 expression in some tumor-infiltrating lymphocytes.

**Table 3:** The relationship between immunohistochemistry expression of Epstein-Barr virus nuclear antigen 1 protein in formalin-fixed paraffin-embedded tumor tissue with the patient clinicopathological variables.

Variable	EBNA-1 (Immunohistochemistry) [n (%)]			
	(-)	(+)	p-value	OR/CI
Age	39.0 ± 10.3	38.0 (31.0 – 45.0)	0.931^a^	0.0 (-5.0-4.0)
Sex				
Male	11/26 (42.3)	81/210 (38.6)	0.713	1.17 (0.54-2.57)
Female	15/26 (57.7)	129/210 (61.4)		
HIV status				
Positive	21/26 (80.8)	149/210 (71.0)	0.360	1.72 (0.64-4.33)
Negative	5/26 (19.2)	61/210 (29.0)		
HIV Status Awareness				
Aware HIV+	16/21 (76.2)	140/149 (94.0)	0.017	0.21 (0.07-0.63)
Unaware HIV+	5/21 (23.8)	9/149 (6.0)		
ART Uptake				
Yes	15/21 (71.4)	129/149 (86.6)	0.099	0.39 (0.14-1.40)
No	6/21 (28.6)	20/149 (13.4)		
CD4 Count	297.7 ± 268.6	220.0 (98.3 – 121.5)	0.734^a^	14.0 (-78.0-117.0)
CD4 Count category				
<200	7/18 (38.9)	62/137 (45.3)	0.609	0.77 (0.30-2.15)
>200	11/18 (61.1)	75/137 (58.7)		
Plasma HIV Viral load	271.9 ± 196.1	0.0 (0.0 – 162.8)	0.068^a^	-121.0 (-252.0-0.0)
Plasma HIV Viral load (c)				
<200	5/9 (55.6)	63/82 (76.8)	0.223^a^	0.38 (0.10-1.34)
>200	4/9 (44.4)	19/82 (23.2)		
Diagnosis				
Invasive OSSN	18/26 (69.2)	148/210 (70.5)	0.896	0.94 (0.41-2.19)
Pre-invasive OSSN	8/26 (30.8)	62/210 (29.5)		
Preinvasive tumor grade				
CIN1	0/8 (0.0)	2/62 (3.2)	0.327^#^	N/A
CIN2	1/8 (12.5)	6/62 (9.7)		
CIN3	1/8 (12.5)	23/62 (37.1)		
CIS	6/8 (75.0)	31/62 (50.0)		
Grouped Preinvasive tumor grade				
CIN1 and II	1/8 (12.5)	8/62 (12.9)	>0.999*	0.96 (0.08-7.61)
CIN3 and CIS	7/8 (87.5)	54/62 (87.1)		
Invasive tumor subtype				
Keratinizing SCC	14/18 (77.8)	142/148 (95.9)	0.002^#^	N/A
Basaloid SCC	2/18 (11.1)	4/148 (2.7)		
Spindle SCC	2/18 (11.1)	2/148 (1.4)		
Invasive tumor grouped				
Keratinizing	14/18 (77.8)	142/148 (95.9)	0.002	0.15 (0.04-0.52)
Non-keratinizing	4/18 (22.2)	6/148 (4.1)		
Grade of invasive tumor				
Well, differentiated	0/18 (0.0)	19/148 (12.8)	0.017^#^	N/A
Moderately differentiated	15/18 (83.3)	122/148 (82.4)		
Poorly differentiated	3/18 (16.7)	7/148 (4.7)		
AJCC Stage				
T1	4/18 (22.2)	32/148 (21.6)	0.772^#^	N/A
T2	6/18 (33.3)	36/148 (24.3)		
T3	7/18 (38.9)	78/148 (52.7)		
T4	1/18 (5.6)	2/148 (1.4)		
Grouped AJCC Stage				
T1/T2	10/18 (55.6)	68/148 (46.0)	0.441	1.47 (0.55-3.94)
T3/T4	8/18 (44.4)	80/148 (54.0)		

^a^Mann-Whitney; *Fishers exact test; ^#^Chisquare trend test. N/A, Not applicable.

PCR for EBV was carried out with multiplex DNA PCR on 178 patient tumor samples with available tissue DNA from frozen samples to confirm the IHC results. **Table 4** summarizes the data for EBV detection in the tissue by multiplex PCR (**Supplement Table S5**) and the association with the clinical and pathologic parameters. Overall, EBV was detected in 143 (80.3%) of the patient tumor samples by PCR. These results were further confirmed using EBNA1 primers (**Supplementary Table S5**) on all the PCR-negative and representative positive patient samples. In total, PCR assays detected EBV DNA in 82.6% of preinvasive (n = 2, 100% CIN 1; n = 3, 100% CIN 2; n = 14, 77.8% CIN 3; and n = 19, 82.6% CIS) and in 79.5% of invasive (n = 98, 79.7% CSCC; n = 5, 83.3% basaloid SCC; and n = 2, 66.7% spindle cell carcinoma) OSSN. PCR detection of EBV DNA was significantly associated with a high HIV viral load (p = 0.022). For invasive OSSN, EBNA1 was expressed in the infiltrating tumor cells, the adjacent preinvasive component, and the adjacent normal-appearing epithelium. One hundred twenty-nine (84.9%) tumors were double-positive for EBV using DNA PCR and EBNA1 IHC. However, 23 (15.1%) tumors were positive for EBNA1 by IHC, but the corresponding frozen tissue tested EBV negative by PCR. Further probing of the IHC slides for these cases found strong staining of tumor nuclei by EBNA1 in the absence of the EBV genome.

**Table 4:** The relationship between Epstein Barr virus detection in tumor using polymerase chain reaction (PCR) with the clinicopathological variables.

Variable	EBV (PCR) (n=%)			
	(-)	(+)	p-value	Estimate (OR/CI)
Age	38.4 ± 10.0	39.0 (32.0-47.0)	0.496	1.00 (-3.00-5.00)
Sex				
Male	14/35 (40.0)	59/143 (41.3)	0.892	0.95 (0.47-1.95)
Female	21/35 (60.0)	84/143 (58.7)		
HIV status				
Positive	25/35 (71.4)	114/143 (79.7)	0.288	1.37 (0.64-1.47)
Negative	10/35 (28.6)	29/143 (20.3)		
HIV Status Awareness				
Aware HIV+	22/25 (88.0)	107/114 (93.9)	0.386^*^	0.47 (0.12-1.82)
Unaware HIV+	3/25 (12.0)	7/114 (6.1)		
ART Uptake				
Yes	22/25 (88.0)	99/114 (86.8)	>0.999^*^	1.11 (0.32-3.87)
No	3/25 (12.0)	15/114 (13.2)		
CD4 Count	290.0 (177.0-417.0)	199.0 (114.0 – 372.0)	0.211^a^	-52.0 (-137.0-34.0)
CD4 Count category				
<200	8/23 (34.8)	50/101 (49.5)	0.250	0.54 (0.21-1.45)
>200	15/23 (65.2)	51/101 (50.5)		
Plasma HIV Viral load	0.0 (0.0 – 0.0)	0.0 (0.0 – 229.3)	0.022^a^	0.0 (0.0-128.0)
Plasma HIV Viral load (c)				
<200	14/15 (93.3)	49/70 (70.0)	0.101^*^	6.00 (0.96-66.4)
>200	1/15 (6.7)	21/70 (30.0)		
Diagnosis				
Invasive OSSN	27/35 (77.1)	105/143 (73.4)	0.653	1.22 (0.51-2.80)
Pre-invasive OSSN	8/35 (22.9)	38/143 (26.6)		
Preinvasive tumor grade				
CIN I	0/8 (0.0)	2/38 (5.3)	0.547^#^	N/A
CIN II	0/8 (0.0)	3/38 (7.9)		
CIN III	4/8 (50.0)	14/38 (36.8)		
CIS	4/8 (50.0)	19/38 (50.0)		
Grouped Preinvasive tumor grade				
CIN I and II	0/8 (0.0)	5/38 (13.2)	0.569^*^	N/A
CIN III and CIS	8/8 (100.0)	33/38 (86.8)		
Invasive tumor subtype				
Keratinizing SCC	25/27 (92.6)	98/105 (93.3)	0.742	N/A
Basaloid SCC	1/27 (3.7)	5/105 (4.8)		
Spindle SCC	1/27 (3.7)	2/105 (1.9)		
Invasive tumor grouped				
Keratinizing	25/27 (92.6)	98/105 (93.3)	0.892	0.89 (0.18-4.47)
Non-keratinizing	2/27 (7.4)	7/105 (6.7)		
Grade of invasive tumor				
Well, differentiated	2/27 (7.4)	15/105 (14.3)	0.492	N/A
Moderately differentiated	23/27 (85.2)	82/105 (78.1)		
Poorly differentiated	2/27 (7.4)	8/105 (7.6)		
AJCC Stage				
T1	5/27 (18.5)	9/105 (8.6)	0.285	N/A
T2	8/27 (29.6)	28/105 (26.7)		
T3	12/27 (44.4)	67/105 (63.8)		
T4	2/27 (7.4)	1/105 (0.9)		
Grouped AJCC Stage				
T1/T2	13/27 (48.1)	37/105 (35.2)	0.217	1.71 (0.70-4.09)
T3/T4	14/27 (52.9)	68/105 (64.8)		

Non-parametric: Confidence interval did use Independent-Samples Hodges-Lehman Median Difference; ^a^Mann-Whitney; *Fishers exact test; ^#^Chisquare trend test. N/A, Not applicable.

A comparison between IHC and EBV PCR showed that EBNA1 IHC had a sensitivity of 84.9% (CI 95%: 0.783–0.897) and a specificity of 52.4% (CI 95%: 0.922–0.983) for predicting EBV positivity using EBV DNA PCR as a reference. The positive and negative predictive value for EBV positivity was 92.8% and 52.4%, respectively (**Supplementary Figure S1**). Our results thus showed that EBV DNA detection by molecular methods and expression of viral proteins was frequent in all stages of preinvasive OSSN and the different subtypes of invasive OSSN.

Viral coinfections (**Table 5**) of EBV and MCPyV were detected by PCR in 23 (12.9%) samples, and EBV and HR-HPV were detected in 12 (6.7%) patient samples. HR-HPV and MCPyV coinfection was detected in 7 (3.9%) patient samples, while all three viruses were detected in only 6 (3.4%) patient samples.

**Table 5:** HPV genotypes detected in tumors by PCR and associated p16 results by diagnostic category.

Patient age	Sex	Histologic Diagnosis	Diagnostic category	p16 expression	HPV genotypes detected
24	M	CIN 3	Preinvasive	–	31,33,59
48	M	CIS	Preinvasive	–	33
35	F	CIS	Preinvasive	–	18, 59
29	F	CIS	Preinvasive	–	18, 59
68	F	Basaloid SCC	Invasive	+	45, 51
55	F	Basaloid SCC	Invasive	–	39
37	M	Basaloid SCC	Invasive	+	33
39	M	Keratinizing SCC	Invasive	–	6
28	F	Keratinizing SCC	Invasive	–	33
48	M	Keratinizing SCC	Invasive	–	33
58	F	Keratinizing SCC	Invasive	–	39
40	M	Keratinizing SCC	Invasive	–	39
35	F	Keratinizing SCC	Invasive	–	58
40	F	Keratinizing SCC	Invasive	+	58
42	M	Keratinizing SCC	Invasive	+	59
53	F	Keratinizing SCC	Invasive	–	18, 39
50	M	Keratinizing SCC	Invasive	–	33, 59

## Discussion

Late presentation or delayed treatment of OSSN puts patients at great risk for visual impairment, disfigurement, and functional immobility, recurrence, metastasis, or death (36–40). Understanding the etiology for OSSN development, especially regarding a potential role for oncogenic viruses, would help create preventative criteria and treatment modalities. Many studies have focused on the role played by HPV in OSSN due to its close association with other SCCs such as carcinoma of the anogenital region and oropharynx (15–17). Globally, studies have reported a variable range of HPV prevalence in OSSN ranging from 0% to 100% (20). A systematic review of 39 studies by Ramberg et al. (20) found a 26% pooled prevalence of HPV in OSSN. Studies from Africa had a lower average prevalence of 15.2% (range 0%–38%) (21–25) compared to countries from other regions, 30.4% (range 0%–100%) (8, 41–71). Other studies did not detect mucosal HPV in cases or controls (8, 22, 42, 60, 68). The low prevalence of HPV reported in previous studies led to the conclusion that it was not associated with OSSN (4). Our results also concur that HPV is not likely the primary cause of OSSN.

In the present study, we prospectively enrolled patients with ocular surface tumors and optimally preserved their tumor samples for investigation *via* PCR and IHC. We evaluated histologically confirmed preinvasive and invasive OSSN for the presence of viral (HPV, EBV, MCPyV, KSHV, and adenovirus) DNA and HR-HPV genotypes and expressed proteins (EBNA1, p16, and MCPyV LT) for HPV, EBV, and MCPyV infection within tumor tissue. Our findings were based on thorough histopathology, IHC, and virus genotyping with a relatively large sample set.

We found a high prevalence of EBV (80.3%) and a low prevalence of HR-HPV (9.0%) and MCPyV (13.5%) by PCR. Yet, there was a low prevalence of HR-HPV detected in both the FFPE and frozen tissue samples. We found EBNA1 expression in 89.0%, p16 overexpression in 4.9%, and MCPyV LT expression in none of the FFPE tissue blocks analyzed using IHC. p16 protein was only overexpressed in invasive OSSN and not in preinvasive OSSN. EBNA1, on the other hand, was expressed in preinvasive (88.6%) and invasive (89.2%) OSSN (**Figure 2**). EBNA1 IHC had a high sensitivity (84.9%) for predicting EBV infection using EBV DNA PCR as a reference.

The finding that women predominated our study population is similar to other studies from Africa (1, 72). Our study population had a median age of 38 years, where interestingly, women were significantly younger than men. The higher frequency of OSSN and the younger age at presentation may be attributed to the higher prevalence of HIV infection among females. In contrast, the age-standardized incidence rates reported by others (73) show evidence that OSSN patients from temperate climates are predominantly older men, with a mean age higher than 60 years (61, 73). The frequency of preinvasive and invasive OSSN was similar in men and women. Our study found that the preinvasive cancer patients were significantly younger than invasive OSSN patients. This finding agrees with the hypothesis that this is the same disease at different stages of progression. We also report the predominance of CIS and CIN3 among the biopsied preinvasive tumors, which could be attributed to early preinvasive lesions sharing similar clinical features with non-OSSN lesions such as pterygium or pinguecula (74). Such patients may not be referred early for specialist treatment and may be treated conservatively using non-surgical means. A review of histologic slides has found that dysplasia is often found in slides previously called as pterygium (75, 76). This suggests that early preinvasive OSSN lesions may be missed if patients only undergo clinical assessment, especially with primary care service providers. Late-stage invasive cancers predominated at primary diagnosis among invasive cancer patients similarly to other late-stage study reports from sub-Saharan Africa (1). In this study, patients with advanced-stage (T3/T4) cancer were significantly older than patients with early-stage (T1/T2) tumors. Showing that patients are presenting late for primary diagnosis. The reasons for delayed diagnosis are unknown but may include patient, community, or service provider factors (77). Studies are needed to determine the factors associated with delayed diagnosis and treatment among OSSN patients in Zambia and Africa.

There is strong evidence that HIV is a significant risk factor for OSSN (78, 79). Our study also found a high prevalence of HIV positivity within our OSSN population (72.8%). We found that 92.1% of the patients with HIV knew their HIV status, and 92.6% were accessing ART with high compliance (99.3%), suggesting that the ART program in Zambia is a success. This is supported by the fact that most patients (74.7%) were virally suppressed. Our study found that low CD4 counts were associated with invasive OSSN, suggesting ART use may help to prevent the development of invasive OSSN. Solar radiation and immunosuppression are major risks factors for OSSN. We found, as in other studies, that OSSN is usually found in only one eye (80).

We found a low frequency of HPV-positive OSSN tumors by PCR, which is consistent with IHC for p16, similarly to previous studies (20). In the present study, p16 was overexpressed in 4.9% of 243 patient samples. In comparison, PCR detected HR-HPV DNA in 9.0% of 178 tissue samples. We detected HR-HPVs in 4 (8.7%) preinvasive OSSN diagnosed as CIN 3 and CIS. PCR did not detect HPV in CIN 1 and CIN 2. The lack of p16 overexpression in the preinvasive tumors by IHC and the lack of HR-HPV detected in CIN 1/2 by PCR suggest that HPV may not play the central role of primary malignant transformation in OSSN.

Our study showed that p16 provided only a low sensitivity for detecting HR-HPV in OSSN; hence, p16 may not be good biomarker for HPV or OSSN. The low sensitivity of p16 for HR-HPV detection may be attributable to the low level of HPV in the ocular tissue. p16 is known to be an excellent biomarker for HPV16 in carcinomas of the cervix and the anogenital region (81, 82). The low frequency of HR-HPV detected in OSSN suggests that only a small portion of these tumors could be HPV associated. Therefore, it became clear that we should consider a role for oncogenic viruses other than HPV in OSSN.

We did not detect KSHV, CMV, or adenoviruses in any OSSN tissue sample analyzed. MCPyV was detected in 13.5% o the patient samples by PCR. However, none of the corresponding FFPE tumor samples expressed MCPyV LT protein by IHC, suggesting that it is not likely relevant to OSSN. MCPyV presents as a ubiquitous infection in which 92% of the general public carries antibodies against it (83). MCPyV sets up a persistent infection in Merkel cells and dendritic cells (84), which might explain why it is detectable by PCR and sometimes IHC. Overall, there is no compelling evidence for MCPyV involvement in OSSN.

We detected a high frequency of EBV genomes (80.3%) and EBNA1 (84.9%) in preinvasive and invasive OSSN. These results suggest that EBV may play a role in OSSN development, particularly in HIV-positive patients. A causal role is supported by the fact that we detected EBV in invasive cancers and all the stages of preinvasive OSSN at very high frequencies. Previously, a few studies have looked at EBV detection in OSSN and have reported seeing EBV in OSSN tissue. Simbiri et al. (85) reported 83% EBV prevalence by PCR in 24 HIV OSSN patients recruited in Botswana, Zambia’s neighboring country (86). Using PCR, a study from Uganda by Galati et al. (87) found significantly higher EBV in OSSN (61.2%) vs. negative control tissue (10.9%). They detected EBV DNA in invasive tumors and all the grades of preinvasive OSSN but did not investigate EBNA1 protein expression in tumor tissues, which our study has done. A study by Woods et al. (65) found the prevalence of EBV DNA in OSSN at only 2.2%, and another study using IHC against latency membrane protein 1 (LMP1) found no EBV in OSSN samples at all (86). The variation in results obtained in previous studies may be attributable to use of assays with different targets and sensitivities as well as differential sample quality.

EBV is a widespread human herpesvirus affecting more than 80% of the adult population and exists as a latent or lytic infection (88). EBV infection has been associated with the development of several types of lymphomas and carcinomas as part of its latent infection (89). EBNA1 is a viral oncoprotein expressed in both latent and lytic replication of EBV infection (84). It is expressed in all forms of latency in proliferating cells and in all EBV-associated tumors, where it is required for the persistence of the EBV genomes. EBNA1 contributes to both the replication and mitotic segregation of EBV episomes and activates the expression of other latency genes essential for cell immortalization and survival in cancer (84). The observation of EBNA1 expression in the infiltrating tumor cells, preinvasive OSSN, and the adjacent normal-appearing epithelium suggests that EBV infection may occur early and play a role in the primary malignant transformation of OSSN. More work is needed to validate our findings, but we feel that our results represent significant progress on this understudied cancer.

While most of our cases, 129 (84.9%), were positive for EBV by IHC and PCR, 23 (15.1%) cases were positive using EBNA1 IHC only but negative by DNA PCR. It is possible that some tissue samples had an inadequate amount of tumor for DNA extraction or that the viral copy number was too low to be detectable. Other researchers have reported intense staining of tumor nuclei by EBNA1 IHC in the absence of EBV genome by PCR in breast cancer specimens (90). Detection of viral protein in a tumor without detection of viral genome is a common occurrence. Similarly, we observed intense staining of tumor nuclei by EBNA1 in the absence of detectable EBV genome. However, we showed that EBNA1 IHC had a high sensitivity for predicting EBV using DNA PCR as a reference. The concordance of EBNA1 IHC and EBV PCR throughout the different tumor stages leads us to consider EBV as the primary oncogenic virus associated with OSSN.

While we found a high prevalence of EBV in preinvasive and invasive OSSN, causation has not been established. Our cohort was made up entirely of OSSN cases because it would not be ethically justified to obtain normal tissue. Non-OSSN cases such as pterygia are inadequate as negative controls, since they share some features with OSSN. Studies establishing a mechanistic role for EBV are needed, but if EBV has a causal role in OSSN, there are existing antiviral therapies that could be tested (91). Overall, our studies support a potential role for EBV in OSSN and that a role for HR-HPVs, CMV, MCPyV, KSHV, or adenovirus appears unlikely. The influence of unknown viral agents still cannot be ruled out. To our knowledge, ours is the first large-scale study to propose EBV as the primary tumor virus associated with OSSN and not HR-HPVs or other tumor viruses reported in previous studies.

## Data Availability Statement

The original contributions presented in the study are included in the article/**Supplementary Material**. Further inquiries can be directed to the corresponding author.

## Ethics Statement

The studies involving human participants were reviewed and approved by the University of Zambia Biomedical Research Ethics Committee (UNZABREC IRB # 015-05-17), the Zambia National Health Research Authority, and the University of Nebraska-Lincoln’s Institutional Review Board (IRB # 20170817442FB). The patients/participants provided their written informed consent to participate in this study.

## Author Contributions

PJ managed the research study at the UTH, provided pathology and IHC expertise, and wrote the first draft of the article. SNS and GH performed the statistical analysis. PM performed all participant recruitment and ophthalmic procedures. FM and TK assisted with pathology work. JS, KP, PG, SS, SK, AE, and KS conducted the molecular work. GK helped perform IHC. DG helped with data entry and analysis. JTW provided on-site laboratory support logistics in Zambia. CW provided logistics and study advice, and PCA conceived of the study, analyzed the results, and helped write and edit the article. All authors contributed to the article and approved the submitted version.

## Funding

This study was funded by the National Institutes of Health (U54CA221204), part of the Zambia AIDS Malignancies Diagnosis and Pathogenesis Program (ZAMDAPP), and by a pilot grant to PCA from the UNMC Cancer Center (P30CA036727).

## Conflict of Interest

The authors declare that the research was conducted in the absence of any commercial or financial relationships that could be construed as a potential conflict of interest.

## Publisher’s Note

All claims expressed in this article are solely those of the authors and do not necessarily represent those of their affiliated organizations, or those of the publisher, the editors and the reviewers. Any product that may be evaluated in this article, or claim that may be made by its manufacturer, is not guaranteed or endorsed by the publisher.

## References

[1] Gichuhi S Sagoo MS Weiss HA Burton MJ. Epidemiology of Ocular Surface Squamous Neoplasia in Africa. Trop Med Int Health (2013) 18:1424–43. doi: 10.1111/tmi.12203 PMC4440345 24237784

[2] Guech-Ongey M Engels EA Goedert JJ Biggar RJ Mbulaiteye SM. Elevated Risk for Squamous Cell Carcinoma of the Conjunctiva Among Adults With AIDS in the United States. Int J Cancer (2008) 122:2590–3. doi: 10.1002/ijc.23384 18224690

[3] UNAIDS. UNAIDS Data 2021 (2021). Available at: https://www.unaids.org/en/resources/documents/2021/2021_unaids_data.

[4] Gurnani B Kaur K Ocular Surface Squamous Neoplasia. [Updated 2021 Dec 20]. In: StatPearls [Internet]. Treasure Island (FL): StatPearls Publishing; 2022 Jan-.Available at: https://www.ncbi.nlm.nih.gov/books/NBK573082/?msclkid=d1cb5b39af5b11eca6b7fe44a03be12c. Ocular Surface Squamous Neoplasia - StatPearls - NCBI Bookshelf (nih.gov).

[5] Starita N Buonaguro L Buonaguro F Tornesello M. Telomerase Promoter Mutations in Human Immunodeficiency Virus-Related Conjunctiva Neoplasia. J Transl Med (2018) 16:77. doi: 10.1186/s12967-018-1456-0 29562930 PMC5861639

[6] Ateenyi-Agaba C Dai M Le Calvez F Katongole-Mbidde E Smet A Tommasino M. TP53 Mutations in Squamous-Cell Carcinomas of the Conjunctiva: Evidence for UV-Induced Mutagenesis. Mutagenesis (2014) 19:399–401. doi: 10.1093/mutage/geh048 15388813

[7] Di Girolamo N Atik A McCluskey PJ Wakefield D. Matrix Metalloproteinases and Their Inhibitors in Squamous Cell Carcinoma of the Conjunctiva. Ocul Surf (2013) 11:193–205. doi: 10.1016/j.jtos.2013.01.006 23838020

[8] Tulvatana W Bhattarakosol P Sansopha L Sipiyarak W Kowitdamrong E Paisuntornsug T. Risk Factors for Conjunctival Squamous Cell Neoplasia: A Matched Case-Control Study. Br J Ophthalmol (2003) 87:396–8. doi: 10.1136/bjo.87.4.396 PMC1771610 12642297

[9] Newton R Ferlay J Reeves G Beral V Parkin DM. Effect of Ambient Solar Ultraviolet Radiation on Incidence of Squamous-Cell Carcinoma of the Eye. Lancet (1996) 347:1450–1. doi: 10.1016/S0140-6736(96)91685-2 8676629

[10] Waddell K Kwehangana J Johnston WT Lucas S Newton R. A Case-Control Study of Ocular Surface Squamous Neoplasia (OSSN) in Uganda. Int J Cancer (2010) 127:427–32. doi: 10.1002/ijc.25040 19908234

[11] Carreira H Coutinho F Carrilho C Lunet N. HIV and HPV Infections and Ocular Surface Squamous Neoplasia: Systematic Review and Meta-Analysis. Br J Cancer (2013) 109:1981–8. doi: 10.1038/bjc.2013.539 PMC3790185 24030075

[12] Angeletti PC Zhang L Wood C. The Viral Etiology of AIDS-Associated Malignancies. Adv Pharmacol (2008) 56:509–57. doi: 10.1016/S1054-3589(07)56016-3 PMC2149907 18086422

[13] Silverberg MJ Abrams DI. AIDS-Defining and non-AIDS-Defining Malignancies: Cancer Occurrence in the Antiretroviral Therapy Era. Curr Opin Oncol (2007) 19:446–51. doi: 10.1097/CCO.0b013e3282c8c90d 17762569

[14] Grogg KL Miller RF Dogan A. HIV Infection and Lymphoma. J Clin Pathol (2007) 60:1365–72. doi: 10.1136/jcp.2007.051953 PMC2095580 18042692

[15] Ji Y Lu H. Malignancies in HIV-Infected and AIDS Patients. Adv Exp Med Biol (2017) 1018:167–79. doi: 10.1007/978-981-10-5765-6_10 29052137

[16] de Martel C Shiels MS Franceschi S Simard EP Vignat J Hall HI. Cancers Attributable to Infections Among Adults With HIV in the United States. AIDS (2015) 29:2173–81. doi: 10.1097/QAD.0000000000000808 PMC4636914 26182198

[17] Park LS Hernández-Ramírez RU Silverberg MJ Crothers K Dubrow R. Prevalence of non-HIV Cancer Risk Factors in Persons Living With HIV/AIDS: A Meta-Analysis. AIDS (2016) 30:273–91. doi: 10.1097/QAD.0000000000000922 PMC4689318 26691548

[18] Ribatti D. The Concept of Immune Surveillance Against Tumors. The First Theories. Oncotarget (2017) 8:7175–80. doi: 10.18632/oncotarget.12739 PMC5351698 27764780

[19] de Martel C Plummer M Vignat J Franceschi S. Worldwide Burden of Cancer Attributable to HPV by Site, Country and HPV Type. Int J Cancer (2017) 141:664–70. doi: 10.1002/ijc.30716 PMC5520228 28369882

[20] Ramberg I Møller-Hansen M Toft PB Funding M Heegaard S. Human Papillomavirus Infection Plays a Role in Conjunctival Squamous Cell Carcinoma: A Systematic Review and Meta-Analysis of Observational Studies. Acta Ophthalmol (2021) 99:478–88. doi: 10.1111/aos.14666 33191633

[21] Ateenyi-Agaba C Franceschi S Wabwire-Mangen F Arslan A Othieno E Binta-Kahwa J. Human Papillomavirus Infection and Squamous Cell Carcinoma of the Conjunctiva. Br J Cancer (2010) 102:262–7. doi: 10.1038/sj.bjc.6605466 PMC2816647 19997105

[22] Ateenyi-Agaba C Weiderpass E Smet A Dong W Dai M Kahwa B. Epidermodysplasia Verruciformis Human Papillomavirus Types and Carcinoma of the Conjunctiva: A Pilot Study. Br J Cancer (2004) 90:1777–9. doi: 10.1038/sj.bjc.6601743 PMC2409740 15150602

[23] Carrilho C Gouveia P Yokohama H Lopes JM Lunet N Ferro J. Human Papillomaviruses in Intraepithelial Neoplasia and Squamous Cell Carcinoma of the Conjunctiva: A Study From Mozambique. Eur J Cancer Prev (2013) 22:566–8. doi: 10.1097/CEJ.0b013e328363005d PMC4167839 23752127

[24] de Koning MN Waddell K Magyezi J Purdie K Proby C Harwood C. Genital and Cutaneous Human Papillomavirus (HPV) Types in Relation to Conjunctival Squamous Cell Neoplasia: A Case-Control Study in Uganda. Infect Agent Cancer (2008) 3:12. doi: 10.1186/1750-9378-3-12 18783604 PMC2551585

[25] Moubayed P Mwakyoma H Schneider DT. High Frequency of Human Papillomavirus 6/11, 16, and 18 Infections in Precancerous Lesions and Squamous Cell Carcinoma of the Conjunctiva in Subtropical Tanzania. Am J Clin Pathol (2004) 122:938–43. doi: 10.1309/T189-UWWV-B71M-9VRC 15539387

[26] Julius P Siyumbwa SN Moonga P Maate F Kaile T Kang G. Clinical and Pathologic Presentation of Primary Ocular Surface Tumors Among Zambians. Ocul Oncol Pathol (2021) 7:108–20. doi: 10.1159/000511610 PMC8024974 33869164

[27] Amin B Gress M,M Meyer Vega D,R, Edge L, Greene SB, Byrd FL. AJCC Cancer Staging Manual. Berlin, Germany: Springer (2018). p. 1032.

[28] Grossniklaus HE Eberhart CG Kivela TT. WHO Classification of Tumours of the Eye 4th Editio. Lyon: International Agency for Research on Cancer (2018).

[29] Samwel K Kahesa C Mwaiselage J Gonzalez D West JT Wood C. Analytical Performance of a Low-Cost Multiplex Polymerase Chain Reaction Human Papillomavirus Genotyping Assay for Use in Sub-Saharan Africa. J Med Virol (2019) 91:308–16. doi: 10.1002/jmv.25329 PMC6519259 30281790

[30] Nishiwaki M Yamamoto T Tone S Murai T Ohkawara T Matsunami T. Genotyping of Human Papillomaviruses by a Novel One-Step Typing Method With Multiplex PCR and Clinical Applications. J Clin Microbiol (2008) 46:1161–8. doi: 10.1128/JCM.00793-07 PMC2292906 18234872

[31] Ko G Cromeans TL Sobsey MD. Detection of Infectious Adenovirus in Cell Culture by mRNA Reverse Transcription-PCR. Appl Environ Microbiol (2003) 69:7377–84. doi: 10.1128/AEM.69.12.7377-7384.2003 PMC309946 14660388

[32] Xu W McDonough MC Erdman DD. Species-Specific Identification of Human Adenoviruses by a Multiplex PCR Assay. J Clin Microbiol (2000) 38:4114–20. doi: 10.1128/JCM.38.11.4114-4120.2000 PMC87550 11060077

[33] Mortazavi Y Lidenge SJ Tran T West JT Wood C Tso FY. The Kaposi’s Sarcoma-Associated Herpesvirus (KSHV) Gh/gL Complex Is the Predominant Neutralizing Antigenic Determinant in KSHV-Infected Individuals. Viruses (2020) 12. doi: 10.3390/v12030256 PMC7150787 32111001

[34] Stevens SJ Vervoort MB van den Brule AJ Meenhorst PL Meijer CJ Middeldorp JM. Monitoring of Epstein-Barr Virus DNA Load in Peripheral Blood by Quantitative Competitive PCR. J Clin Microbiol (1999) 37:2852–7. doi: 10.1128/JCM.37.9.2852-2857.1999 PMC85394 10449464

[35] Ayee R Ofori MEO Tagoe EA Languon S Searyoh K Armooh L. Genotypic Characterization of Epstein Barr Virus in Blood of Patients With Suspected Nasopharyngeal Carcinoma in Ghana. Viruses (2020) 12. doi: 10.3390/v12070766 PMC7412455 32708700

[36] Miller CV Wolf A Klingenstein A Decker C Garip A Kampik A. Clinical Outcome of Advanced Squamous Cell Carcinoma of the Conjunctiva. Eye (Lond) (2014) 28:962–7. doi: 10.1038/eye.2014.79 PMC4135264 24858526

[37] Karcioglu ZA Toth J. Relation Between P53 Overexpression and Clinical Behavior of Ocular/Orbital Invasion of Conjunctival Squamous Cell Carcinoma. Ophthal Plast Reconstr Surg (2000) 16:443–9. doi: 10.1097/00002341-200011000-00008 11106189

[38] Merz LE Afriyie O Jiagge E Adjei E Foltin SK Ludwig ML. Clinical Characteristics, HIV Status, and Molecular Biomarkers in Squamous Cell Carcinoma of the Conjunctiva in Ghana. Heal Sci Rep (2019) 2:e108. doi: 10.1002/hsr2.108 PMC6375545 30809594

[39] Desai SJ Pruzan NL Geske MJ Jeng BH Bloomer MM Vagefi MR. Local and Regional Spread of Primary Conjunctival Squamous Cell Carcinoma. Eye Contact Lens (2018) 44 Suppl 1:S312–5. doi: 10.1097/ICL.0000000000000264 27058828

[40] McKelvie PA Daniell M McNab A Loughnan M Santamaria JD. Squamous Cell Carcinoma of the Conjunctiva: A Series of 26 Cases. Br J Ophthalmol (2002) 86:168–73. doi: 10.1136/bjo.86.2.168 PMC1770993 11815342

[41] Adachi W. Human Papilloma Virus in the Conjunctiva in Ocular Surface Diseases. Jpn J Clin Ophthalmol (1995) 49:439.

[42] Guthoff R Marx A Stroebel P. No Evidence for a Pathogenic Role of Human Papillomavirus Infection in Ocular Surface Squamous Neoplasia in Germany. Curr Eye Res (2009) 34:666–71. doi: 10.1080/02713680903007162 19899994

[43] Jung S-M Lin H-C Chu P-H Wu H-H Shiu T-F Huang SL. Expression of Cell Cycle-Regulatory Proteins, MIB-1, P16, P53, and P63, in Squamous Cell Carcinoma of Conjunctiva: Not Associated With Human Papillomavirus Infection. Virchows Arch (2006) 448:301–5. doi: 10.1007/s00428-005-0104-2 16328355

[44] Karcioglu ZA Issa TM. Human Papilloma Virus in Neoplastic and Non-Neoplastic Conditions of the External Eye. Br J Ophthalmol (1997) 81:595 LP – 8. doi: 10.1136/bjo.81.7.595 PMC1722242 9290377

[45] Kuo K-T Chang H-C Hsiao C-H Lin M-C. Increased Ki-67 Proliferative Index and Absence of P16INK4 in CIN-HPV Related Pathogenic Pathways Different From Cervical Squamous Intraepithelial Lesion. Br J Ophthalmol (2006) 90:894–9. doi: 10.1136/bjo.2005.086314 PMC1857176 16540490

[46] Lauer SA Malter JS Meier JR. Human Papillomavirus Type 18 in Conjunctival Intraepithelial Neoplasia. Am J Ophthalmol (1990) 110:23–7. doi: 10.1016/s0002-9394(14)76932-6 2164326

[47] Manderwad GP Kannabiran C Honavar SG Vemuganti GK. Lack of Association of High-Risk Human Papillomavirus in Ocular Surface Squamous Neoplasia in India. Arch Pathol Lab Med (2009) 133:1246–50. doi: 10.5858/133.8.1246 19653719

[48] McDonnell JM McDonnell PJ Mounts P Wu T-C Green WR. Demonstration of Papillomavirus Capsid Antigen in Human Conjunctival Neoplasia. Arch Ophthalmol (1986) 104:1801–5. doi: 10.1001/archopht.1986.01050240075043 3024607

[49] McDonnell JM Mayr AJ Martin WJ. DNA of Human Papillomavirus Type 16 in Dysplastic and Malignant Lesions of the Conjunctiva and Cornea. N Engl J Med (1989) 320:1442–6. doi: 10.1056/NEJM198906013202202 2541337

[50] McDonnell JM McDonnell PJ Sun YY. Human Papillomavirus DNA in Tissues and Ocular Surface Swabs of Patients With Conjunctival Epithelial Neoplasia. Invest Ophthalmol Vis Sci (1992) 33:184–9. 1309728

[51] Moyer AB Roberts J Olsen RJ Chévez-Barrios P. Human Papillomavirus-Driven Squamous Lesions: High-Risk Genotype Found in Conjunctival Papillomas, Dysplasia, and Carcinoma. Am J Dermatopathol (2018) 40:486–90. doi: 10.1097/DAD.0000000000001139 29533279

[52] Afrogheh AH Jakobiec FA Hammon R Grossniklaus HE Rocco J Lindeman NI. Evaluation for High-Risk HPV in Squamous Cell Carcinomas and Precursor Lesions Arising in the Conjunctiva and Lacrimal Sac. Am J Surg Pathol (2016) 40:519–28. doi: 10.1097/PAS.0000000000000581 26735858

[53] Nagarajan P El-Hadad C Gruschkus SK Ning J Hudgens CW Sagiv O. PD-L1/PD1 Expression, Composition of Tumor-Associated Immune Infiltrate, and HPV Status in Conjunctival Squamous Cell Carcinoma. Invest Ophthalmol Vis Sci (2019) 60:2388–98. doi: 10.1167/iovs.19-26894 PMC6890426 31141610

[54] Nakamura Y Mashima Y Kameyama K Mukai M Oguchi Y. Detection of Human Papillomavirus Infection in Squamous Tumours of the Conjunctiva and Lacrimal Sac by Immunohistochemistry, *In Situ* Hybridisation, and Polymerase Chain Reaction. Br J Ophthalmol (1997) 81:308–13. doi: 10.1136/bjo.81.4.308 PMC1722165 9215061

[55] Palazzi MA Erwenne CM Villa LL. Detection of Human Papillomavirus in Epithelial Lesions of the Conjunctiva. Sao Paulo Med J (2000) 118:125–30. doi: 10.1590/s1516-31802000000500003 PMC11175550 11018845

[56] Peralta R Valdivia A Estañol P Villegas V Pimienta C Treviño E. Low Frequency of Human Papillomavirus Infection in Conjunctival Squamous Cell Carcinoma of Mexican Patients. Infect Agent Cancer (2011) 6:24. doi: 10.1186/1750-9378-6-24 22099431 PMC3226560

[57] Ramberg I Toft PB Georgsen JB Siersma VD Funding M Jensen DH. Conjunctival Intraepithelial Neoplasia and Carcinoma: Distinct Clinical and Histological Features in Relation to Human Papilloma Virus Status. Br J Ophthalmol (2021) 105:878–83. doi: 10.1136/bjophthalmol-2019-315011 31649051

[58] Revollo B Videla S Sirera G García-Cuyás F Parés D Corral J. Natural History of Anal Squamous Intraepithelial Lesions in HIV-Positive Men With Normal Baseline Cytology. AIDS Patient Care STDS (2019) 33:459–65. doi: 10.1089/apc.2019.0186 31682165

[59] Scott IU Karp CL Nuovo GJ. Human Papillomavirus 16 and 18 Expression in Conjunctival Intraepithelial Neoplasia. Ophthalmology (2002) 109:542–7. doi: 10.1016/s0161-6420(01)00991-5 11874759

[60] Sen S Sharma A Panda A. Immunohistochemical Localization of Human Papilloma Virus in Conjunctival Neoplasias: A Retrospective Study. Indian J Ophthalmol (2007) 55:361–3. doi: 10.4103/0301-4738.33822 PMC2636007 17699945

[61] Shrestha T Choi W Kim GE Yang JM Yoon KC. Human Papilloma Virus Identification in Ocular Surface Squamous Neoplasia by P16 Immunohistochemistry and DNA Chip Test: A Strobe-Compliant Article. Med (Baltimore) (2019) 98:e13944–4. doi: 10.1097/MD.0000000000013944 PMC6336645 30633172

[62] Tabrizi SN McCurrach FE Drewe RH Borg AJ Garland SM Taylor HR. Human Papillomavirus in Corneal and Conjunctival Carcinoma. Aust N Z J Ophthalmol (1997) 25:211–5. doi: 10.1111/j.1442-9071.1997.tb01394.x 9296295

[63] Asadi-Amoli F Heidari AB Jahanzad I Jabbarvand M. Detection of Human Papillomavirus in Squamous Cell Carcinoma of Conjunctiva by Nested PCR: A Case Control Study in Iran. Acta Med Iran (2011) 49:707–14. 22131239

[64] Toth J Karcioglu ZA Moshfeghi AA Issa TM Al-Ma’ani JR Patel KV. The Relationship Between Human Papillomavirus and P53 Gene in Conjunctival Squamous Cell Carcinoma. Cornea (2000) 19:159–62. doi: 10.1097/00003226-200003000-00007 10746446

[65] Woods M Chow S Heng B Glenn W Whitaker N Waring D. Detecting Human Papillomavirus in Ocular Surface Diseases. Invest Ophthalmol Vis Sci (2013) 54:8069–78. doi: 10.1167/iovs.13-13140 24255045

[66] Auw-Haedrich C Martin G Spelsberg H Sundmacher R Freudenberg N Maier P. Expression of P16 in Conjunctival Intraepithelial Neoplasia Does Not Correlate With HPV-Infection. Open Ophthalmol J (2008) 2:48–56. doi: 10.2174/1874364100802010048 19516893 PMC2687927

[67] Chauhan S Sen S Sharma A Dar L Kashyap S Kumar P. Human Papillomavirus: A Predictor of Better Survival in Ocular Surface Squamous Neoplasia Patients. Br J Ophthalmol (2012) 96:1517–21. doi: 10.1136/bjophthalmol-2012-301907 22942158

[68] Dushku N Hatcher SLS Albert DM Reid TW. P53 Expression and Relation to Human Papillomavirus Infection in Pingueculae, Pterygia, and Limbal Tumors. Arch Ophthalmol (1999) 117:1593–9. doi: 10.1001/archopht.117.12.1593 10604662

[69] Eng H-L Lin T-M Chen S-Y Wu S-M Chen W. Failure to Detect Human Papillomavirus DNA in Malignant Epithelial Neoplasms of Conjunctiva by Polymerase Chain Reaction. Am J Clin Pathol (2002) 117:429–36. doi: 10.1309/RVUP-QMU3-5X6W-3CQ1 11888082

[70] Galor A Garg N Nanji A Joag M Nuovo G Palioura S. Human Papilloma Virus Infection Does Not Predict Response to Interferon Therapy in Ocular Surface Squamous Neoplasia. Ophthalmology (2015) 122:2210–5. doi: 10.1016/j.ophtha.2015.07.007 PMC4624006 26337001

[71] Griffin H Mudhar HS Rundle P Shiraz A Mahmood R Egawa N. Human Papillomavirus Type 16 Causes a Defined Subset of Conjunctival *in Situ* Squamous Cell Carcinomas. Mod Pathol Off J United States Can Acad Pathol Inc (2020) 33:74–90. doi: 10.1038/s41379-019-0350-5 PMC6930848 31485010

[72] Steele KT Steenhoff AP Bisson GP Nkomazana O. Ocular Surface Squamous Neoplasia Among HIV-Infected Patients in Botswana. SAMJ South Afr Med J (2015) 105:379–83. doi: 10.7196/SAMJ.8254 26242668

[73] Nagaiah G Stotler C Orem J Mwanda WO Remick SC. Ocular Surface Squamous Neoplasia in Patients With HIV Infection in Sub-Saharan Africa. Curr Opin Oncol (2010) 22:437–42. doi: 10.1097/CCO.0b013e32833cfcf9 PMC4209293 20639761

[74] Hirst LW Axelsen RA Schwab I. Pterygium and Associated Ocular Surface Squamous Neoplasia. Arch Ophthalmol (Chicago Ill 1960) (2009) 127:31–2. doi: 10.1001/archophthalmol.2008.531 19139334

[75] Zoroquiain P Jabbour S Aldrees S Villa N Bravo-Filho V Dietrich H. High Frequency of Squamous Intraepithelial Neoplasia in Pterygium Related to Low Ultraviolet Light Exposure. Saudi J Ophthalmol Off J Saudi Ophthalmol Soc (2016) 30:113–6. doi: 10.1016/j.sjopt.2016.02.007 PMC4908070 27330387

[76] Mejía L Zapata M Gil J. An Unexpected Incidence of Ocular Surface Neoplasia on Pterygium Surgery. A Retrospective Clinical and Histopathological Report. Cornea (2020) 40(8):1002–6. doi: 10.1097/ICO.0000000000002586 33201056

[77] Gichuhi S Kabiru J M’bongo Zindamoyen A Rono H Ollando E Wachira J. Delay Along the Care-Seeking Journey of Patients With Ocular Surface Squamous Neoplasia in Kenya. BMC Health Serv Res (2017) 17:485. doi: 10.1186/s12913-017-2428-4 28705204 PMC5512725

[78] Nkomazana O Tshitswana D. Ocular Complications of HIV Infection in Sub-Sahara Africa. Curr HIV/AIDS Rep (2008) 5:120–5. doi: 10.1007/s11904-008-0019-z 18627660

[79] Ateenyi-Agaba C. Conjunctival Squamous-Cell Carcinoma Associated With HIV Infection in Kampala, Uganda. Lancet (Lond Eng) (1995) 345:695–6. doi: 10.1016/s0140-6736(95)90870-6 7885126

[80] Chisi SK Kollmann MKH Karimurio J. Conjunctival Squamous Cell Carcinoma in Patients With Human Immunodeficiency Virus Infection Seen at Two Hospitals in Kenya. East Afr Med J (2006) 83:267–70. doi: 10.4314/eamj.v83i5.9432 16866221

[81] Singh S Mittal R Ghosh A Tripathy D Rath S. High-Resolution Anterior Segment Optical Coherence Tomography in Intraepithelial Versus Invasive Ocular Surface Squamous Neoplasia. Cornea (2018) 37:1292–8. doi: 10.1097/ICO.0000000000001680 29985794

[82] O’Neill CJ McCluggage WG. P16 Expression in the Female Genital Tract and its Value in Diagnosis. Adv Anat Pathol (2006) 13:8–15. doi: 10.1097/01.pap.0000201828.92719.f3 16462152

[83] Carter JJ Paulson KG Wipf GC Miranda D Madeleine MM Johnson LG. Association of Merkel Cell Polyomavirus-Specific Antibodies With Merkel Cell Carcinoma. J Natl Cancer Inst (2009) 101:1510–22. doi: 10.1093/jnci/djp332 PMC2773184 19776382

[84] Frappier L. The Epstein-Barr Virus EBNA1 Protein. Sci (Cairo) (2012) 2012:438204. doi: 10.6064/2012/438204 PMC3820569 24278697

[85] Simbiri KO Murakami M Feldman M Steenhoff AP Nkomazana O Bisson G. Multiple Oncogenic Viruses Identified in Ocular Surface Squamous Neoplasia in HIV-1 Patients. Infect Agent Cancer (2010) 5:6. doi: 10.1186/1750-9378-5-6 20346104 PMC2859758

[86] Hamed-Azzam S Edison N Briscoe D Frenkel S Mukari A Strauss M. Role of Oncogenic Viruses in the Development Ocular Surface Squamous Neoplasia. Int Ophthalmol (2021) 41:3599–605. doi: 10.1007/s10792-021-01933-8 34173153

[87] Galati L Combes JD Gupta P Sen R Robitaille A Brancaccio RN. Detection of a Large Spectrum of Viral Infections in Conjunctival Premalignant and Malignant Lesions. Int J Cancer (2020) 147:2862–70. doi: 10.1002/ijc.33149 32525572

[88] Smatti MK Al-Sadeq DW Ali NH Pintus G Abou-Saleh H Nasrallah GK. Epstein-Barr Virus Epidemiology, Serology, and Genetic Variability of LMP-1 Oncogene Among Healthy Population: An Update. Front Oncol (2018) 8:211. doi: 10.3389/fonc.2018.00211 29951372 PMC6008310

[89] Ayee R Ofori MEO Wright E Quaye O. Epstein Barr Virus Associated Lymphomas and Epithelia Cancers in Humans. J Cancer (2020) 11:1737–50. doi: 10.7150/jca.37282 PMC7052849 32194785

[90] Murray PG Lissauer D Junying J Davies G Moore S Bell A. Reactivity With A Monoclonal Antibody to Epstein-Barr Virus (EBV) Nuclear Antigen 1 Defines a Subset of Aggressive Breast Cancers in the Absence of the EBV Genome. Cancer Res (2003) 63:2338–43. 12727860

[91] Jiang L Xie C Lung HL Lo KW Law G-L Mak N-K. EBNA1-Targeted Inhibitors: Novel Approaches for the Treatment of Epstein-Barr Virus-Associated Cancers. Theranostics (2018) 8:5307–19. doi: 10.7150/thno.26823 PMC6276081 30555548

