# Supplementary material for: Epstein–Barr Virus, But Not Human Papillomavirus, Is Associated With Preinvasive and Invasive Ocular Surface Squamous Neoplasias in Zambian Patients
Source: Front Oncol. 2022 Apr 14;12:864066. doi: 10.3389/fonc.2022.864066 (PMC9047892; doi:10.3389/fonc.2022.864066)
Supplement: Supplementary file 1 [file DataSheet_1.docx]

**Supplement Tables and Figures**

Supplement Table 1: The relation between the immunohistochemistry expression of p16 in formalin-fixed paraffin-embedded tumor tissue with clinicopathological variables.

| Variable | P16 (Immunohistochemistry) (n (%)) | | |  |
| --- | --- | --- | --- | --- |
|  | **(-)** | **(+)** | **p-value** | **OR** |
| Age | 38.0 (31.0 – 45.0) | 42.3 ± 11.3 | 0.227^a^ | -3.0 (-2.0-10.0) |
| Sex |  |  |  |  |
| - Male | 88/231 (38.1) | 6/12 (50.0) | 0.409 | 0.62 (0.19-2.03) |
| - Female | 143/231 (61.9) | 6/12 (50.0) |  |  |
| HIV status |  |  |  |  |
| - Positive | 168/231 (72.7) | 9/12 (75.0) | >0.999^*^ | 0.89 (0.25-3.41) |
| - Negative | 63/231 (27.3) | 3/12 (25.0) |  |  |
| HIV Status Awareness |  |  |  |  |
| - Aware HIV+ | 154/168 (91.7) | 9/9 (100.0) | >0.999^*^ | N/A |
| - Unaware HIV+ | 14/168 (8.3) | 0/9 (0.0) |  |  |
| ART Uptake |  |  |  |  |
| - Yes | 145/168 (86.3) | 6/9 (66.7) | 0.129^*^ | 3.13 (0.81-11.6) |
| - No | 23/168 (13.7) | 3/9 (33.3) |  |  |
| CD4 Count | 242.5 (125.0 – 418.3) | 47.0 (25.8 – 249.3) | 0.012^a^ | -147.0 (339.0-30.0) |
| CD4 Count category |  |  |  |  |
| - <200 | 66/154 (42.9) | 4/6 (66.7) | 0.405^*^ | 0.38 (0.07-1.66) |
| - >200 | 88/154 (57.1) | 2/6 (33.3) |  |  |
| Plasma HIV Viral load | 0.0 (0.0 – 191.5) | 0.0 (0.0 – 113.0) | 0.565^a^ | N/A |
| Plasma HIV Viral load (c) |  |  |  |  |
| - <200 | 68/92 (73.9) | 3/3 (100.0) | 0.569^*^ | N/A |
| - >200 | 24/92 (26.1) | 0/3 (0.0) |  |  |
| Diagnosis |  |  |  |  |
| - Invasive OSSN | 159/231 (68.8) | 12/12 (100.0) | 0.020^*^ | 0.00 (0.00-0.75) |
| - Pre-invasive OSSN | 72/231 (31.2) | 0/12(0.0) |  |  |
| Preinvasive tumor grade |  |  |  |  |
| - CIN-1 | 2/72 (2.8) | 0/0 (0.0) | N/A | N/A |
| - CIN-2 | 8/72 (11.1) | 0/0 (0.0) |  |  |
| - CIN-3 | 25/72 (34.7) | 0/0 (0.0) |  |  |
| - CIS | 37/72 (51.4) | 0/0 (0.0) |  |  |
| Grouped Preinvasive tumor grade |  |  |  |  |
| - CIN 1 and 2 | 10/72 (13.9) | 0/0 (0.0) | N/A | N/A |
| - CIN 3 and CIS | 62/72 (86.1) | 0/0 (0.0) |  |  |
| Invasive tumor subtype |  |  |  |  |
| - Keratinizing SCC | 151/159 (95.0) | 10/12 (83.3) | 0.383^#^ | N/A |
| - Basaloid SCC | 4/159 (2.5) | 2/12 (16.7) |  |  |
| - Spindle SCC | 4/159 (2.5) | 0/12 (0.0) |  |  |
| Invasive tumor grouped |  |  |  |  |
| - Keratinizing | 151/159 (95.0) | 10/12 (83.3) | 0.148^*^ | 3.8 (0.7-16.7) |
| - Non-keratinizing | 8/159 (5.0) | 2/12 (16.7) |  |  |
| Grade of invasive tumor |  |  |  |  |
| - Well, differentiated | 21/159 (13.2) | 0/12 (0.0) | <0.001^#^ | N/A |
| - Moderately differentiated | 132/159 (83.0) | 8/12 (66.7) |  |  |
| - Poorly differentiated | 6/159 (3.8) | 4/12 (33.3) |  |  |
| AJCC Stage |  |  |  |  |
| - T1 | 37/159 (23.3) | 1/12 (8.3) | 0.272^#^ | N/A |
| - T2 | 39/159 (24.5) | 3/12 (25.0) |  |  |
| - T3 | 80/159 (50.3) | 8/12 (66.7) |  |  |
| - T4 | 3/159 (1.9) | 0/12 (0.0) |  |  |
| Grouped AJCC Stage |  |  |  |  |
| - T1/T2 | 76/159 (47.8) | 4/12 (33.3) | 0.383^*^ | 1.83 (0.58-5.64) |
| - T3/T4 | 83/159 (52.2) | 8/12 (66.7) |  |  |

# Chi-square trend; * Fisher’s exact test; ^a^ Mann Whitney test

Supplement Table 2: The relationship between human papillomavirus infection status of the tumor using polymerase chain reaction with the clinicopathological variables.

| **Variable** | **HR-HPV (PCR) (n=%)** | |  |  |
| --- | --- | --- | --- | --- |
|  | **(-)** | **(+)** | **p-value** | **OR/CI** |
| Age | 38.5 (32.0 - 46.0) | 43.1±12.0 | 0.215^a^ | 4.0 (-2.0-10.0) |
| Sex |  |  |  |  |
| - Male | 66/162 (40.7) | 7/16 (43.8) | 0.815 | 0.88 (0.33-2.32) |
| - Female | 96/162 (59.3) | 9/16 (56.2) |  |  |
| HIV status |  |  |  |  |
| - Positive | 128/162 (79.0) | 11/16 (68.8) | 0.344 | 1.71 (0.62-4.84) |
| - Negative | 34/162 (21.0) | 5/16 (31.2) |  |  |
| HIV Status Awareness |  |  |  |  |
| - Aware HIV+ | 120/128 (93.8) | 9/11 (81.8) | 0.180^*^ | 3.33 (0.63-14.8) |
| - Unaware HIV+ | 8/128 (6.2) | 2/11 (18.2) |  |  |
| ART Uptake |  |  |  |  |
| - Yes | 112/128 (87.5) | 9/11 (81.8) | 0.635^*^ | 1.56 (0.31-7.18) |
| - No | 16/128 (12.5) | 2/11 (18.2) |  |  |
| CD4 Count | 223.5 (119.8 – 390.8) | 220.8 ± 190.2 | 0.439^a^ | -47.5 (-191.0-72.0) |
| CD4 Count category |  |  |  |  |
| - <200 | 53/116 (45.7) | 5/8 (62.5) | 0.472^*^ | 0.50 (0.13-2.05) |
| - >200 | 63/116 (54.3) | 3/8 (37.5) |  |  |
| Plasma HIV Viral load | 0.0 (0.0 – 184.5) | 0.0 (0.0 – 505.5) | >0.999^a^ | 0.0 (-215.0 – 505.0) |
| Plasma HIV Viral load (c) |  |  |  |  |
| - <200 | 53/116 (45.7) | 5/8 (62.5) | >0.472^*^ | 0.5 (0.1 - 2.1) |
| - >200 | 63/116 (54.3) | 3/8 (37.5) |  |  |
| Diagnosis |  |  |  |  |
| - Invasive OSSN | 120/162 (74.1) | 12/16 (75.0) | >0.999^*^ | 0.95 (0.32-2.81) |
| - Pre-invasive OSSN | 42/162 (25.9) | 4/16 (25.0) |  |  |
| Preinvasive tumor grade |  |  |  |  |
| - CIN-1 | 2/42 (4.8) | 0/4 (0.0) | 0.284^#^ | N/A |
| - CIN-2 | 3/42 (7.1) | 0/4 (0.0) |  |  |
| - CIN-3 | 17/42 (40.5) | 1/4 (25.0) |  |  |
| - CIS | 20/42 (47.6) | 3/4 (75.0) |  |  |
| Grouped Preinvasive tumor grade |  |  |  |  |
| - CIN 1 and 2 | 5/42 (11.9) | 0/4 (0.0) | >0.999^*^ | N/A |
| - CIN 3 and CIS | 37/42 (88.1) | 4/4 (100.0) |  |  |
| Invasive tumor subtype |  |  |  |  |
| - Keratinizing SCC | 114/120 (95.0) | 9/12 (75.0) | 0.106^#^ | N/A |
| - Basaloid SCC | 3/120 (2.5) | 3/12 (25.0) |  |  |
| - Spindle SCC | 3/120 (2.5) | 0/12 (0.0) |  |  |
| Invasive tumor grouped |  |  |  |  |
| - Keratinizing | 114/120 (95.0) | 9/12 (75.0) | 0.036^*^ | 6.33 (1.50-26.7) |
| - Non-keratinizing | 6/120 (5.0) | 3/12 (25.0) |  |  |
| Grade of invasive tumor |  |  |  |  |
| - Well, differentiated | 16/120 (13.3) | 1/12 (8.3) | 0.270^#^ | N/A |
| - Moderately differentiated | 96/120 (80.0) | 9/12 (75.0) |  |  |
| - Poorly differentiated | 8/120 (6.7) | 2/12 (16.7) |  |  |
| AJCC Stage |  |  |  |  |
| - T1 | 13/120 (10.8) | 1/12 (8.3) | 0.279^#^ | N/A |
| - T2 | 34/120 (28.3) | 2/12 (16.7) |  |  |
| - T3 | 71/120 (59.2) | 8/12 (66.7) |  |  |
| - T4 | 2/120 (1.7) | 1/12 (8.3) |  |  |
| Grouped AJCC Stage |  |  |  |  |
| - T1/T2 | 47/120 (39.2) | 3/12 (25.0) | 0.534^*^ | 1.93 (0.54-6.87) |
| - T3/T4 | 73/120 (60.8) | 9/12 (75.0) |  |  |

# Chi-square trend; * Fisher’s exact test; ^a^ Mann Whitney test

Supplement Table 3: The relationship between the Merkel cell virus infection status of the tumor using polymerase chain reaction with the clinicopathological variables.

| Variable | MCV (n (%)) | | |  |
| --- | --- | --- | --- | --- |
|  | **(-)** | **(+)** | **p-value** | **OR** |
| Age | 39.3 ± 10.7 | 39.5 (35.5-45.5) | 0.377^a^ | 2.0 (-2.0-6.0) |
| Sex |  |  |  |  |
| - Male | 63/154 (40.9) | 10/24 (42.1) | 0.944 | 0.97 (0.40-2.34) |
| - Female | 91/154 (59.1) | 14/24 (58.3) |  |  |
| HIV status |  |  |  |  |
| - Positive | 119/154 (77.3) | 20/24 (83.3) | 0.605^*^ | 0.68 (0.24-1.97) |
| - Negative | 35/154 (22.7) | 4/24 (16.7) |  |  |
| HIV Status Awareness |  |  |  |  |
| - Aware HIV+ | 111/119 (93.3) | 18/20 (90.0) | 0.637^*^ | 1.5 (0.31-7.43) |
| - Unaware HIV+ | 8/119 (6.7) | 2/20 (10.0) |  |  |
| ART Uptake |  |  |  |  |
| - Yes | 103/119 (86.6) | 18/20 (90.0) | >0.999^*^ | 0.72 (0.15-3.16) |
| - No | 16/119 (13.44) | 2/20 (10.0) |  |  |
| CD4 Count | 199.5 (116.8-384.0) | 406.4 ± 289.8 | 0.063^a^ | 129.5 (-10.0-251.0) |
| CD4 Count category |  |  |  |  |
| - <200 | 55/110 (50.0) | 3/14 (21.4) | 0.051 | 3.67 (1.02-12.7) |
| - >200 | 55/110 (50.0) | 11/14 (78.6) |  |  |
| Plasma HIV Viral load | 0.0 (0.0-200.0) | 0.0 (0.0-181.0) | 0.664^a^ | 0.0 (-115.0-0.0) |
| Plasma HIV Viral load (c) |  |  |  |  |
| - <200 | 57/78 (73.1) | 6/7 (85.7) | 0.671 | 0.45 (0.04-3.17) |
| - >200 | 21/78 (26.9) | 1/7 (14.3) |  |  |
| Diagnosis |  |  |  |  |
| - Invasive OSSN | 113/154 (73.4) | 19/24 (79.2) | 0.547^*^ | 0.73 (0.28-2.09) |
| - Pre-invasive OSSN | 41/154 (26.6) | 5/24 (20.8) |  |  |
| Preinvasive tumor grade |  |  |  |  |
| - CIN-1 | 2/41 (4.9) | 0/5 (0.0) | 0.096^#^ | N/A |
| - CIN-2 | 1/41 (2.4) | 2/5 (40.0) |  |  |
| - CIN-3 | 17/41 (41.5) | 1/5 (20.0) |  |  |
| - CIS | 21/41 (51.2) | 2/5 (40.0) |  |  |
| Grouped Preinvasive tumor grade |  |  |  |  |
| - CIN 1 and 2 | 3/41 (7.3) | 2/5 (40.0) | 0.084^*^ | 0.12 (0.02-0.94) |
| - CIN 3 and CIS | 38/41 (92.7) | 3/5 (60.0) |  |  |
| Invasive tumor subtype |  |  |  |  |
| - Keratinizing SCC | 106/113 (93.8) | 17/19 (89.5) | 0.850^*^ | N/A |
| - Basaloid SCC | 4/113 (3.5) | 2/19 (10.5) |  |  |
| - Spindle SCC | 3/113 (2.7) | 0/19 (0.0) |  |  |
| Invasive tumor grouped |  |  |  |  |
| - Keratinizing | 106/113 (93.8) | 17/19 (89.5) | 0.488^*^ | 1.78 (0.35-8.10) |
| - Non-keratinizing | 7/113 (6.2) | 2/19 (10.5) |  |  |
| Grade of invasive tumor |  |  |  |  |
| - Well, differentiated | 15/113 (13.3) | 2/19 (10.5) | 0.578^#^ | N/A |
| - Moderately differentiated | 90/113 (79.6) | 15/19 (78.9) |  |  |
| - Poorly differentiated | 8/113 (7.1) | 2/19 (10.5) |  |  |
| AJCC Stage |  |  |  |  |
| - T1 | 14/113 (12.4) | 0/19 (0.0) | N/A | N/A |
| - T2 | 32/113 (28.3) | 4/19 (21.1) |  |  |
| - T3 | 64/113 (56.6) | 15/19 (78.9) |  |  |
| - T4 | 3/113 (2.7) | 0/19 (0.0) |  |  |
| Grouped AJCC Stage |  |  |  |  |
| - T1/T2 | 46/113 (40.7) | 4/19 (21.1) | 0.102^*^ | 2.58 (0.82-7.46) |
| - T3/T4 | 67/113 (59.3) | 15/19 (78.9) |  |  |

# Chi-square trend; * Fisher’s exact test; ^a^ Mann Whitney test

Supplement Table 4**:** The relationship between independent variables.

|  |  |  | **Statistical probability** | **OR/CL** |
| --- | --- | --- | --- | --- |
| Age vs. Sex | Male | 40.6 ± 9.90 | P=0.007a | 4.0 (1.0-6.0) |
|  | Female | 37.0 (29.0-43.0) |  |  |
| Age vs. HIV status | Positive | 39.0 ± 9.4 | P=0.007^a^ | -4.0 (-7.0-(-1.0) |
|  | Negative | 35.0 (27.0-43.3) |  |  |
| Age vs diagnosis | Preinvasive | 35.8 ± 10.2 | P=0.003^a^ | -5.0 (-7.0-(-2.0)) |
|  | Invasive | 39.0 (34.0-46.0) |  |  |
| Age vs diagnosis | Non-Keratinizing | 42.0 (34.0-48.0) | P=0.534^a^ | 2.0 (-5.0-10.0) |
|  | Keratinizing | 39.0 (34.0-46.0) |  |  |
| Age vs Stage | T1/T2 | 37.0 (31.0-44.0) | P=0.005^a^ | -4.0 (-7.0-(-1.0) |
|  | T3/T4 | 42.0 (35.0-48.0) |  |  |
| Age vs CD4 C | <200 | 39.0 (34.0-46.0) | P=0.598^a^ | -1.0 (-4.0-2.0) |
|  | >200 | 38.9 ± 9.1 |  |  |
| Age vs HIV Viral Load C | <200 | 41.7 ± 9.3 | P=0.003^b^ | -6.7 (-11.0-(-2.3)) |
|  | >200 | 35.0 ± 9.0 |  |  |
| HIV status vs. Sex | HIV- Male | 27/66 (40.9) | P=0.664 | 1.1 (0.6-2.0) |
|  | HIV- Female | 39/66 (59.1) |  |  |
|  | HIV+ Male | 67/177 (37.9) |  |  |
|  | HIV+ Female | 110/177 (62.1) |  |  |
| HIV status vs. diagnosis | HIV- Preinvasive | 24/66 (36.4) | P=0.160 | 1.2 (0.7-2.1) |
|  | HIV- Invasive | 42/66 (63.6) |  |  |
|  | HIV+ Preinvasive | 48/177 (27.1) |  |  |
|  | HIV+ Invasive | 129/177 (72.9) |  |  |
| HIV status vs. diagnosis | HIV- Keratinizing | 2/42 (4.8) | P=0.730 | 0.8 (0.2-3.3) |
|  | HIV- Non-keratinizing | 40/42 (95.2) |  |  |
|  | HIV+ Keratinizing | 8/129 (6.2) |  |  |
|  | HIV+ non-keratinizing | 121/129 (93.8) |  |  |
| HIV status | HIV- T1/T2 | 26/42 (61.9) | P=0.024 | 2.3 (1.1-4.6) |
|  | HIV- T3/T4 | 16/42 (38.1) |  |  |
|  | HIV+ T1/T2 | 54/129 (41.9) |  |  |
|  | HIV+ T3/T4 | 75/129 (58.1) |  |  |
| CD4 C | <200, Preinvasive | 15/70 (21.4) | P=0.071 | 0.52 (0.2-1.1) |
|  | <200, Invasive | 55/70 (78.6) |  |  |
|  | >200, Preinvasive | 31/90 (34.4) |  |  |
|  | >200, Invasive | 59/90 (65.6) |  |  |
| CD4 C | <200, T1/T2 | 21/55 (38.2) | P=0.413 | 0.73 (0.4-1.5) |
|  | <200, T3/T4 | 34/55 (61.8) |  |  |
|  | >200, T1/T2 | 27/59 (45.8) |  |  |
|  | >200, T3/T4 | 32/59 (54.2) |  |  |
| CD4 count | Preinvasive | 325.0 (136.0-524.0) | P=0.025^a^ | -79.0 (-165.0-(-8.0)) |
|  | Invasive | 203.5 (115.5-388.3) |  |  |
| CD4count | T1/T2 | 213.0 (141.3-406.5) | P=0.230^a^ | -41.0 (-98.0-27.0) |
|  | T3/T4 | 184.0 (96.5-384.8) |  |  |
| HIV Viral load C | <200, Preinvasive | 13/71 (18.3) | P<0.001 | 0.16 (0.06-0.5) |
|  | <200, Invasive | 58/71 (81.7) |  |  |
|  | >200, Preinvasive | 14/24 (58.3) |  |  |
|  | >200, Invasive | 10/24 (41.7) |  |  |
| HIV Viral load C | <200, T1/T2 | 17/58 (29.3) | P<0.001^*^ | 0.0 (0.0-0.2) |
|  | <200, T3/T4 | 41/58 (70.7) |  |  |
|  | >200, T1/T2 | 10/10 (100) |  |  |
|  | >200, T3/T4 | 0/10 (0.0) |  |  |
| HIV Viral load | Preinvasive | 195.0 (0.0-443.0) | P=0.002^a^ | -76.0 (-224.0-0.0) |
|  | Invasive | 0.0 (0.0-119.5) |  |  |
| HIV Viral load | T1/T2 | 65.0 (0.0-284.0) | P=0.001^a^ | 0.0 (-128.0-0.0) |
|  | T3/T4 | 0.0 (0.0-0.0) |  |  |

C = categorical; * = fishers exact; a = Mann-Whitney test; b= Student t-test

Supplement Table 5: Primers used for beta-globin, multiplex DNA tumor viruses, EBV confirmation, and multiplex HPV amplification.

| **Gene Target** | **Primer Name** | **Primer sequence** | **Amplicon Size** |
| --- | --- | --- | --- |
| Beta globin | B-globin F | (ACACAACTGTGTTCACTAGC) | 119 bp |
|  | B-globin R | (CAACTTCATCCACGTTCACC) |  |
| **DNATV multiplex** | | | |
| KS | Forward | 5’-AGCCGAAAGATTCCACCAT-3’ | 233 bp |
|  | Reverse | 5’-TCCGTGTTGTCTACGTCCAG-3’ |  |
| EBV | Forward | 5’-CCCCAGATCACGGCTGTCTA-3’ | 186 bp |
|  | Reverse | 5’-TCGCACCCCACAGCATAAAC-3’ |  |
| MCV2 | Forward | 5’-CACACGGGACCAACTCAAGA-3’ | 313 bp |
|  | Reverse | 5’-TCGCAGAAGAGATCCTCCCA-3’ |  |
| ADENO2 | Forward | 5’-TCAGACACGGTTTCGTCAGG-3’ | 482 bp |
|  | Reverse | 5’-CGCTCGAGAATGAAAGTGCG-3’ |  |
| **EBV Confirmation Primers** | | | |
| EBNA-1 | QP1 | 5’-GCCGGTGTGTTCGTATATGG-3’ | 213 bp |
|  | QP2 | 5’-CAAAACCTCAGCAAATATATGAG-3’ |  |
