# Supplementary figures and images for: Epstein–Barr Virus, But Not Human Papillomavirus, Is Associated With Preinvasive and Invasive Ocular Surface Squamous Neoplasias in Zambian Patients
Source: Front Oncol. 2022 Apr 14;12:864066. doi: 10.3389/fonc.2022.864066 (PMC9047892; doi:10.3389/fonc.2022.864066)

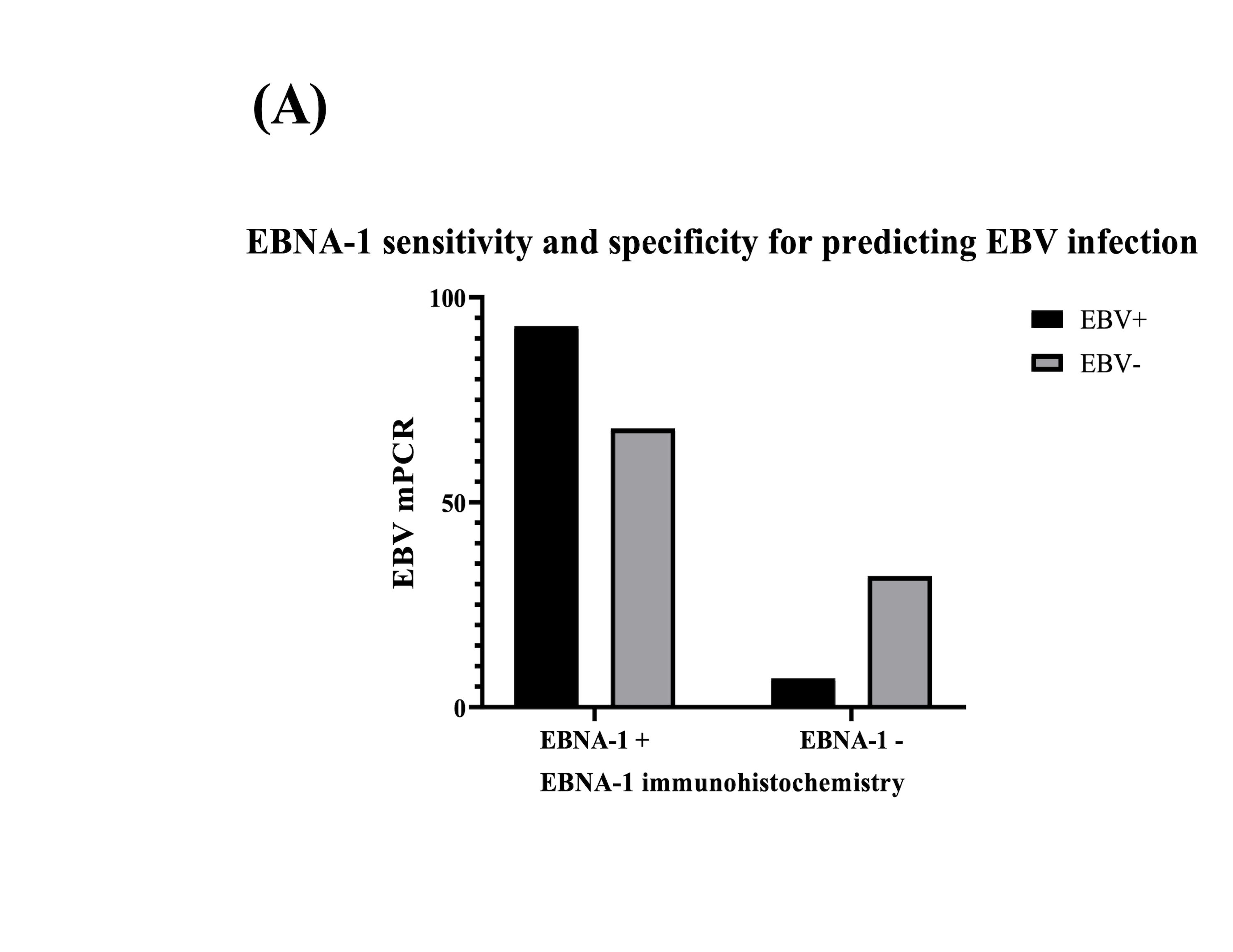

Supplement: Supplementary file 2 [file Image_1.tiff]

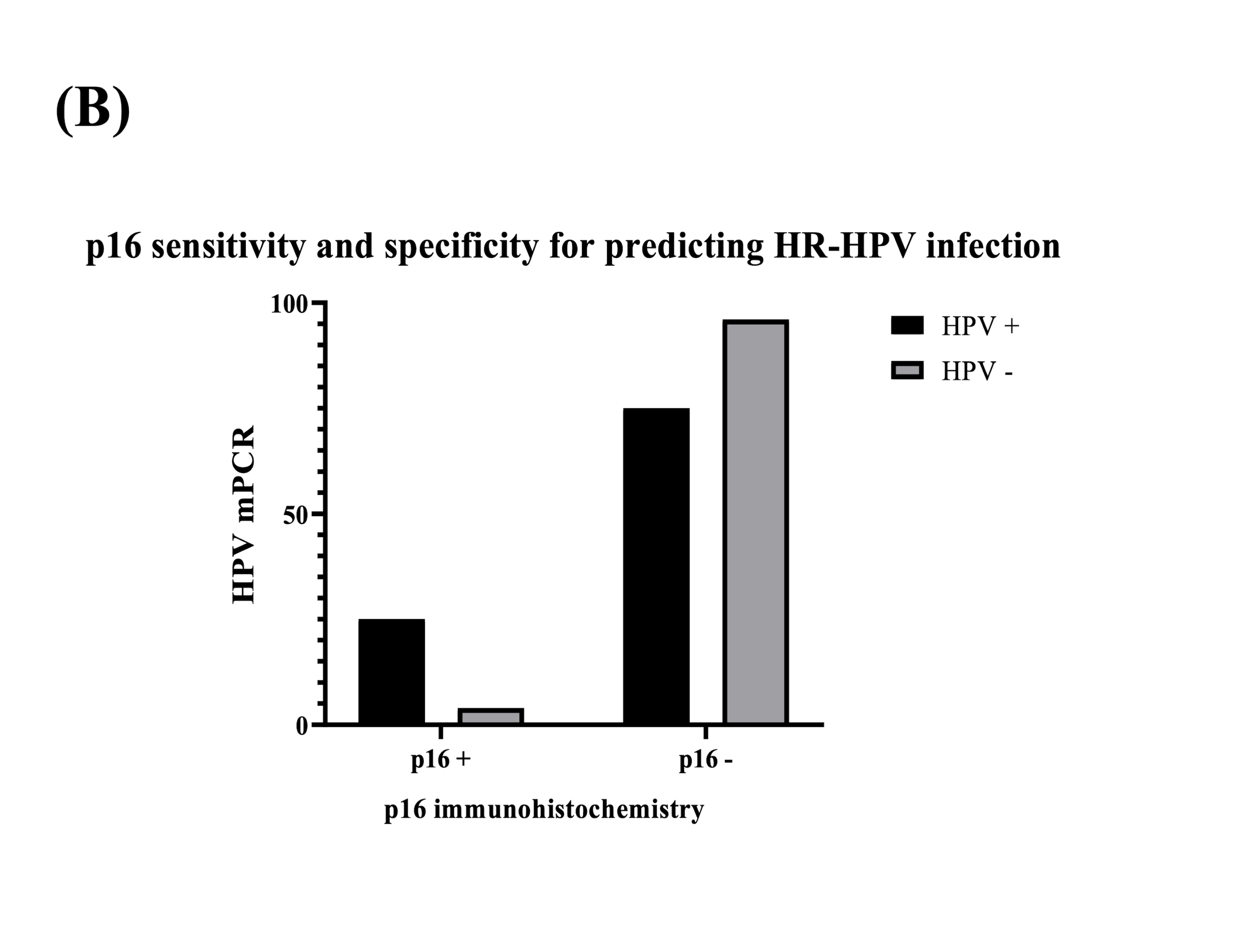

Supplement: Supplementary file 3 [file Image_2.tiff]

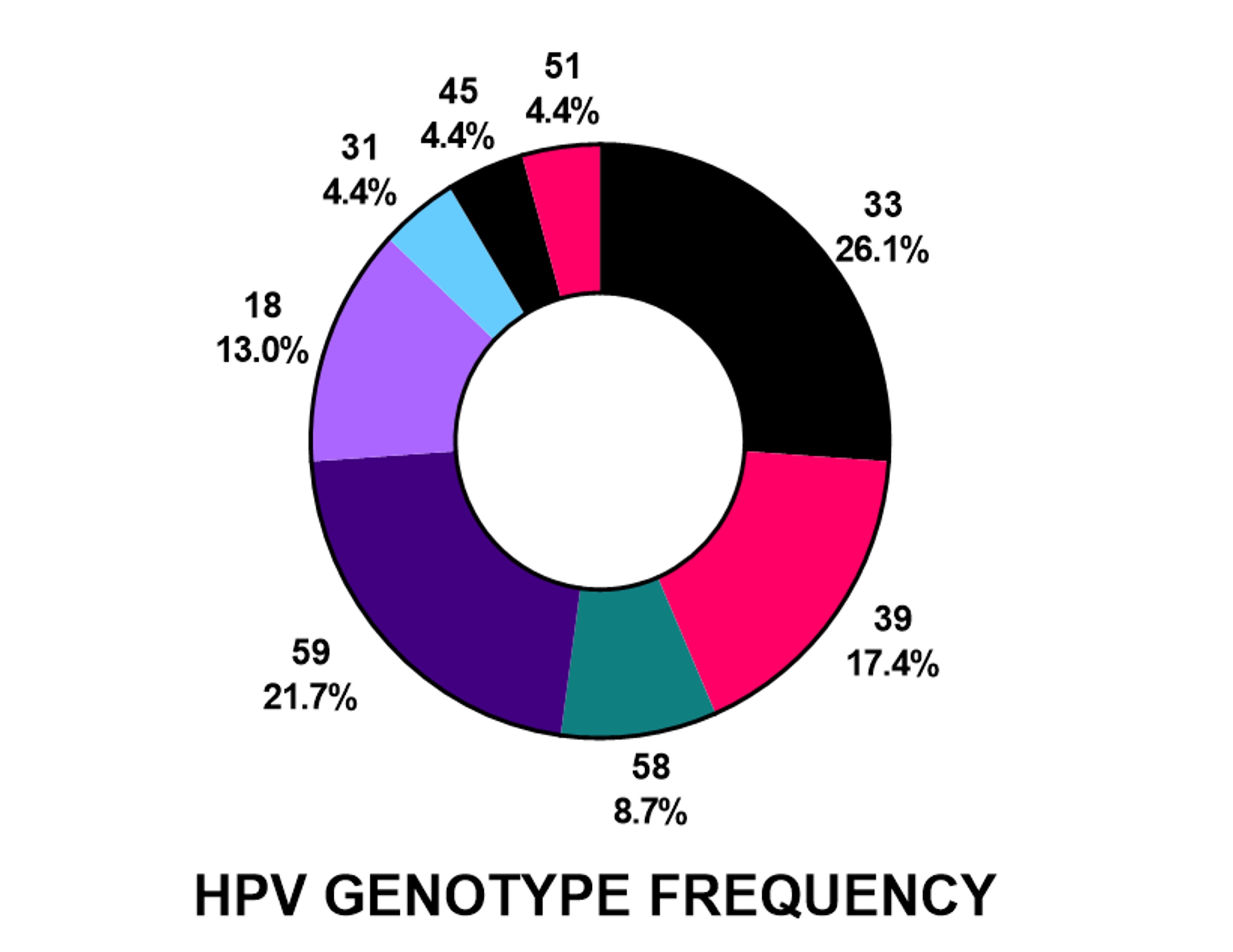

Supplement: Supplementary file 4 [file Image_3.tiff]
